# Metabonomic analysis of the anti-hepatic fibrosis effect of *Ganlong capsules*

**DOI:** 10.3389/fphar.2023.1122118

**Published:** 2023-03-23

**Authors:** ChangLing Lv, YinRui Li, Ling Ou, Jie Zhou, Fang Peng, DingYu Wu

**Affiliations:** ^1^ College of Pharmacy, Dali University, Dali, China; ^2^ Yunnan Provincial Key Laboratory of Entomological Biopharmaceutical R&D, Dali, China; ^3^ Department Of Pharmacy, Mengzi People’s Hospital, Mengzi, China

**Keywords:** Ganlong capsule, hepatic fibrosis, CCl_4_, metabolomic analysis, LC-MS

## Abstract

**Context:** Hepatic fibrosis is a progressive condition, often attributed to metabolic disorders, which may promote cirrhosis and liver cancer. *Ganlong capsules* derived from *Periplaneta Americana* have been shown to have a therapeutic effect on liver fibrosis but little is known about the molecular mechanisms involved.

**Objective:** To investigate the metabolic modulations produced by *Ganlong capsules* in liver fibrosis.

**Methods:** A carbon tetrachloride- (CCl_4_) treated rat model of liver fibrosis was constructed and *Ganlong capsules* administered. Levels of serum liver enzymes and pathological changes to the liver were evaluated. Non-targeted metabolomics of liver, serum and urine were used to investigate metabolic regulatory mechanisms.

**Results:** *Ganlong capsules* reduced serum levels of liver enzymes and improved pathological changes in the rat model of fibrosis. Non-targeted metabolomics showed that *Ganlong capsules* ameliorated pathways of glycerophospholipid, linoleic acid, pyrimidine, glycine, butyric acid, valine, serine, threonine and arachidonic acid metabolism and biosynthesis of leucine and isoleucine. Such pathways influence the development of CCl_4_-induced liver fibrosis.

**Conclusion:** *Ganlong capsules* had an anti-fibrotic hepatoprotective effect and regulated lipid, butyric acid, amino acid and arachidonic acid metabolism.

## Introduction

Abnormal hyperplasia of liver connective tissue leads to hepatic fibrosis (HF). Interstitial or protofibril extracellular matrix (ECM) accumulates, causing scarring and pathological changes to hepatic structure and function (Cao, et al., 2018; Parola and Pinzani, 2019; Cheng, et al., 2021; Khurana, et al., 2021).

Timely treatment of liver fibrosis may reduce the morbidity rate of cirrhosis and progression to liver cancer (Yeoh, Barton, et al., 2011), indicating the urgent need to identify anti-fibrosis agents (Ni, et al., 2016).

Metabolomics or metabonomics allows the quantification of metabolites which largely consist of small molecules with relative molecular masses of <1,000 to illuminate the relationship with pathophysiology. This is a holistic, systematic and dynamic approach to the study of metabolic changes in response to internal and external factors with utility for diagnostic and pharmacological efficacy assessments (Stringer, et al., 2016; Beyoglu and Idle, 2020).

The complex pathogenesis of liver fibrosis has made therapeutic management difficult but some Chinese herbal medicines have anti-fibrotic effects with few toxic side effects (Shan et al., 2019).

*Periplaneta americana* has been much used in traditional Chinese medicine to strengthen the spleen, invigorate blood circulation, reduce swelling, promote granulation and as a diuretic. It is used in the clinic to treat digestive tract diseases, chronic heart failure, skin lesions and periodontitis (Li, et al., 2018; Zeng, et al., 2019).

*Periplaneta americana* extract is the principal active ingredient of *Ganlong capsules,* marketed in China for the treatment of hepatitis B. *Ganlong capsules* have been shown to inhibit hepatic stellate cell (HSC) activity *in vitro* and *in vivo* and to protect the liver from damage caused by chronic alcohol consumption or pig serum-induced immune fibrosis in rats (Yong, 2017; Yong L. Y. 2018; Yong S. M. 2018; Shao and Yang, 2018; Yong C. L. 2019; Yong G. C. 2019; Yong W. H. 2019).

Thus, the effects of *Ganlong capsules* in ameliorating various acute and chronic liver diseases, in addition to hepatitis B, have been characterized but anti-fibrotic mechanisms remain poorly understood.

Metabolomic studies of Chinese medicine remain undeveloped, illustrating the need to apply this approach to *Ganlong capsules,* allowing for the complexities of composition and pharmacological mechanism.

The current study sought to identify the anti-fibrosis target of *Ganlong capsules* and clarify the mechanism of action. A rat model of liver fibrosis was generated by CCl_4_ treatment and *Ganlong capsules* given by gavage. Non-targeted metabolomics was performed to examine changes in liver tissue, serum and urine metabolites*.*

## Materials and methods

### Reagents

*Ganlong capsules* were purchased from Saino Pharmaceutical Co., Ltd., colchicine tablets from Yunnan Phytology Co., LTD. and aminotransferase (AST), alanine aminotransferase (ALT) assay kits from Nanjing Jiancheng Biological Engineering Institute.

Laminin (LN), type III procollagen (PC III), hyaluronic acid (HA) and type IV collagen (Col-IV) were assayed in liver tissue by ELISA kit (Nanjing Jiancheng Bioengineering Institute, Nanjing, China). CCl_4_ was purchased from Tianjin Fuchen Chemical Reagent Factory (Lot No: 2021030), Voila extra virgin olive oil from yihai grain and oil industry co., LTD. (Lot No: 20201205), ammonium acetate (Lot No: 631-61-8) from SIGMA and methanol (Lot No: 67-56-1) and acetonitrile (Lot No: 75-05-8) from CNW.

### Animals

Six-week-old Sprague-Dawley (SD) male rats (160–200 g) were purchased from Hunan Jingda Experimental Animal Co., Ltd. (No. SXCK 2019–0004) and acclimated to a temperature of 25°C ± 2°C with a 12 h light/12 h dark cycle for 3 days prior to experiments.

Ethical approval was granted by the Experimental Animal Ethics Committee of Dali University.

### Induction of rat model of liver fibrosis

CCL_4_ diluted with olive oil to give a 40% solution was given at a dose of 1 mL/kg body weight by intraperitoneal injection twice a week for 8 weeks.

### Animal grouping and dosing regimen

Thirty-six rats were randomly divided into two groups: normal control (*n* = 6) and model (*n* = 30). Model rats received CCl_4_ and control rats an equal volume of solvent by intraperitoneal injection twice a week for 9 weeks. Liver fibrosis was ascertained by hematoxylin-eosin (HE) staining of liver samples.

After confirmation of successful modelling, model rats were randomly divided into five groups (*n* = 6): *Ganlong capsules* high-dose (250 mg/kg), medium-dose (125 mg/kg) and low-dose (62.5 mg/kg), colchicine (0.2 mg/kg) and model (untreated).

Colchicine and *Ganlong capsules* were given at a dose of 0.5 mL/100 g by intragastric administration while model and control rats received an equal volume of normal saline by intragastric administration once a day for 4 weeks. Rat body weights were recorded at 2 days intervals for 13 weeks. Urine was collected from fasting rats during 12 h of darkness following the final treatment administration and blood collected from the abdominal aorta after anesthetization. Livers and spleens were extracted and weighed for calculation of the viscera index (%) by the following formula: viscera weight (g)/body weight (g) × 100%.

Rats were euthanized by intraperitoneal injection of 300 mg per kg pentobarbital sodium, according to the animal euthanasia guidelines of the Experimental Animal Ethics Committee of Dali University.

### Biochemical analyses

Blood was left to stand at room temperature for 2 h and centrifuged at 3, 000 rpm for 10 min to obtain serum. Hepatic function was assessed by measurement of serum ALT and AST by kit, according to the manufacturer’s instructions.

### ELISA assay

Hyaluronic acid (HA), laminin (LN), type III procollagen (PC III) and type IV collagen (Col-IV) in liver tissue were detected by ELISA kit, according to the manufacturer’s instructions.

### HE and Masson’s trichrome staining

One mm^3^ of rat liver was fixed with 4% tissue cell fixative, embedded in paraffin and sectioned. HE and Masson staining were performed and observed under an inverted microscope at ×200 magnification to score the degree of liver fibrosis.

### Untargeted metabolomics of serum, urine and liver tissue

Differential metabolites in serum, urine and liver tissue from control, model and *Ganlong capsules* treated groups were screened by liquid chromatography-mass spectrometry (LC-MS).

### Sample preparation

100 μl serum or urine was transferred to EP tubes, 400 μl extraction solution (equal volumes of methanol and acetonitrile) added with vortex mixing for 30 s. Samples were sonicated in ice water for 10 min, left to stand at −40°C for 60 min and centrifuged at 12,000 rpm at 4°C for 20 min.

25 mg liver tissue was added to 500 μl extraction solution (methanol: acetonitrile: water at 2:2:1 (V/V), containing isotope-labeled internal standard mixture), ground for 4 min (35 Hz) and sonicated for 5 min in an ice water bath. The above steps were repeated 3 times. Samples were incubated in a water bath at 40°C for 1 h and centrifuged at 12,000 rpm at 4°C for 20 min. Supernatants were used for analysis and quality control samples prepared by mixing equal amounts of supernatant from all samples.

### Liquid chromatography-mass spectrometry

Target compounds were separated by Vanquish UPLC with an ACQUITY UPLC HSS T3 1.8 μm 2.1 × 100 mm liquid chromatography column. The liquid chromatographic A phase contained 25 mmoL/L ammonia and 25 mmoL/ammonium acetate and the B phase acetonitrile. LC-MS was performed, according to the instrument operation manual.

### Data processing and analysis

UHPLC-Q Exactive mass spectrometry information was visualized using R language, peak area data retained for all groups with no more than 50% null values, normalized (using internal standard) and missing values simulated (minimum value of one-half).

Multivariate analysis of pre-processed data was performed by SIMCA-P 16.0 and unsupervised principal component analysis (PCA) applied to asses system stability and repeatability and distribution among samples, followed by supervised mode orthogonal partial least squares analysis (OPLA). Sample metabolic profiles and differential metabolites were evaluated by partial least squares-discrimination analysis (OPLS-DA), verified by response permutation testing (RPT) to identify “overfitting”. Fold change (FC), variable important in projection (VIP) and *p*-values were visualized by the OPLS-DA model with FC > 1.2 or FC < 0.8 and VIP >1. Values of FC < 0.8, VIP >1 and *p* < 0.05 were used to screen differential metabolites considered as potential biomarkers.

Differential metabolites were also identified using the human metabolome database HMDB (http://www.hmdb.ca/) and the KEGG database (http://www.kegg.jp/kegg/pathway.html).The MetaboAnalyst 5.0 website (http://www.metaboanalyst.ca/) was used to perform metabolic pathway analysis and clustering heat mapping on the basis of Raw *p* < 0.05.

### Statistical analysis

All data are expressed as mean ± SD for at least three independent experiments. Prism 9.0 software (GraphPad Software, United States) was used for statistical analysis and comparisons between groups assessed by one-way ANOVA. A value of *p* < 0.05 was regarded as statistically significant.

## Results

### Effect of *Ganlong capsules* on CCl_4_-induced liver fibrosis

Liver index, spleen index, body weight, levels of liver fibrotic markers, PC III, LN, Hyp, Col-IV, HA, activities of AST, and ALT and pathological changes in the liver were all measured 13 weeks after administration of *Ganlong capsules*.

**FIGURE 1:**
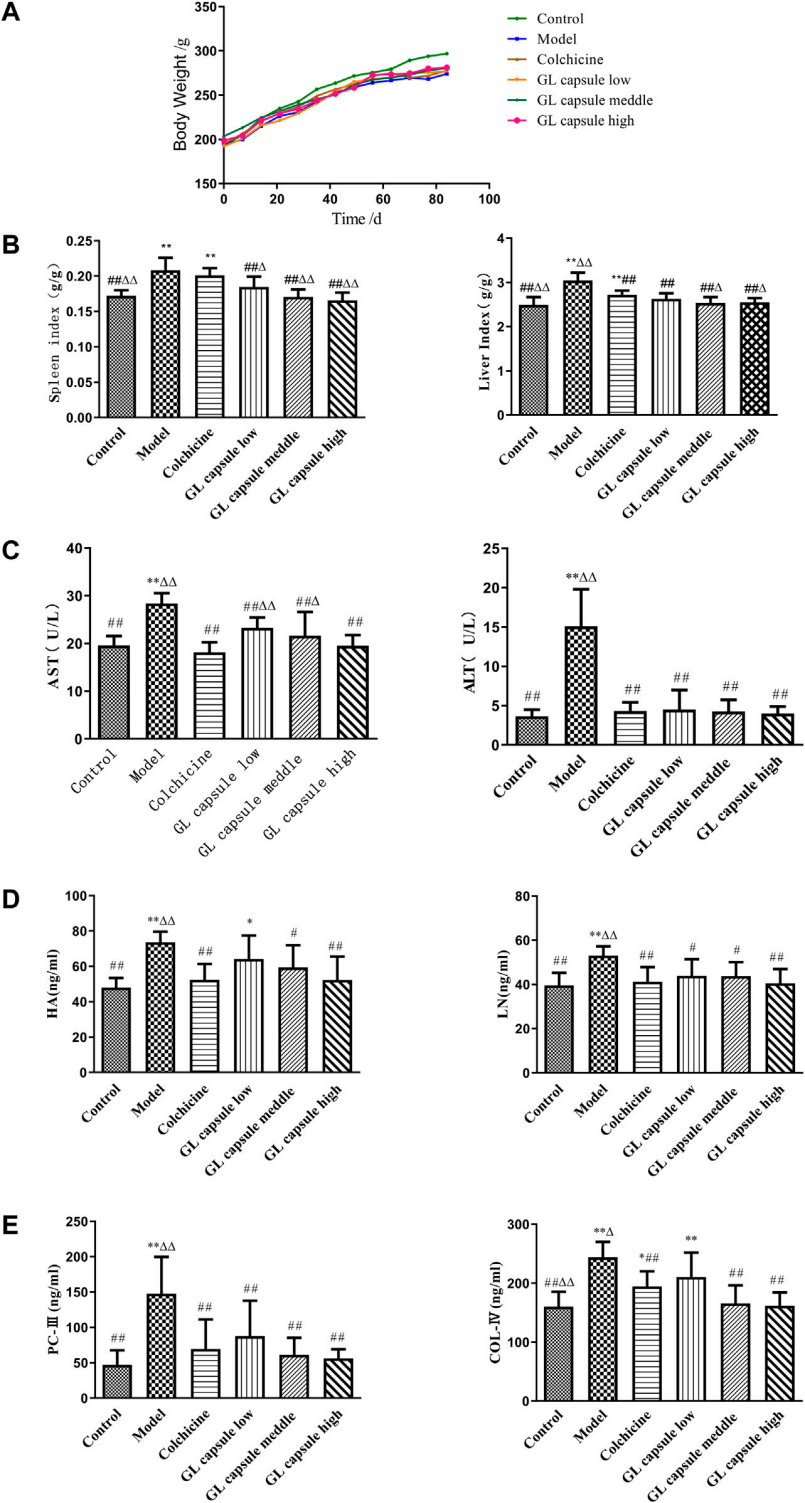
Ganlong capsule treatment increases body weight and liver index. **(A)** Body weight of the six groups. **(B)** Liver index and spleen index of the six groups. **(C)** Effects of Ganlong capsule ALT and AST levels in serum. **(D,E)** Effect of Ganlong capsules on serum HA, LN, PC-III and COL-IV contents in rats with liver fibrosis. Control group, model group, Ganlong capsule low group, Ganlong capsule medium group and Ganlong capsule high group (*n*=6 in each group). Compared with normal control group, *p < 0.05, ^**^ *p* < 0.01; compared with model group, ^#^ *p* < 0.05, ^##^ *p* < 0.01; compared with colchicine group, ^Δ^ *p* < 0.05, ^ΔΔ^ *p* < 0.01.

Control rats gained weight at non-statistically significantly higher rate than rats in other groups (Figure 1A) and CCl_4_ treatment resulted in significantly higher liver and spleen indexes, although values were reduced by *Ganlong capsules* (*p* < 0.01, Figure 1B).

ALT and AST activities were higher in fibrosis model rats (*p* < 0.01) and administration of *Ganlong capsules* reduced the degree of liver fibrosis (*p* < 0.01, Figure 1C) and downregulated markers of hepatic fibrosis, including Hyp, HA, LN, PC III, and Col-IV (Figures 1D, E).

**FIGURE 2:**
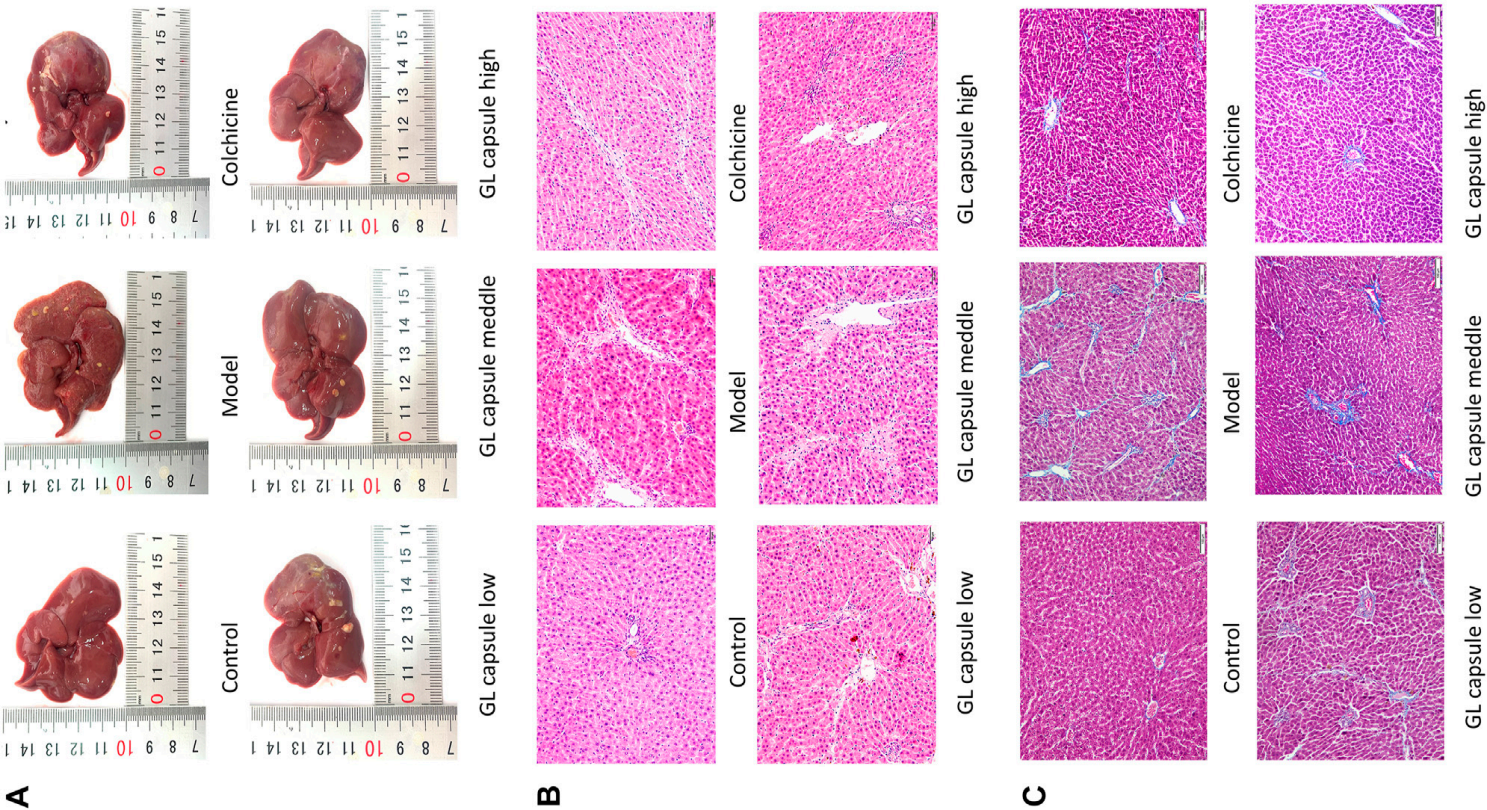
General morphological and pathological evaluation of liver tissues. **(A)** Morphological effects of Ganlong capsules on the appearance of liver of rats with liver fibrosis. **(B)** Effect of Ganlong capsules on HE pathological histology of liver tissue in hepatic fibrosis rats (200×). **(C)** Effect of Ganlong capsules on Masson pathological histology of liver tissues of rats with liver fibrosis (200×) Control group, model group, Ganlong capsule low group, Ganlong capsule medium group and Ganlong capsule high group (*n* = 6 in each group). Compared with normal control group, ^*^ *p* < 0.05, ^**^ *p* < 0.01; compared with model group, ^#^ *p* < 0.05, ^##^ *p* < 0.01; compared with colchicine group, ^∆^ *p* < 0.05, ^∆∆^ *p* < 0.01.

General liver morphology and Masson and HE staining indicated that liver cells of control rats were closely and radially arranged with few inflammatory cells but enlarged confluent area, obvious inflammatory cell infiltration, widely spaced collagen fibres and disorganized lobular structure and hepatocyte arrangement were all present in fibrosis model rats (Figure 2A).

Treatment with either colchicine and *Ganlong capsules* ameliorated liver damage (Figure 2B) and reduced total collagen deposition, decreased fibrotic lesions and normalized lobular structures (Figure 2C).

### Multivariate analysis of liver tissue metabolomics

**FIGURE 3:**
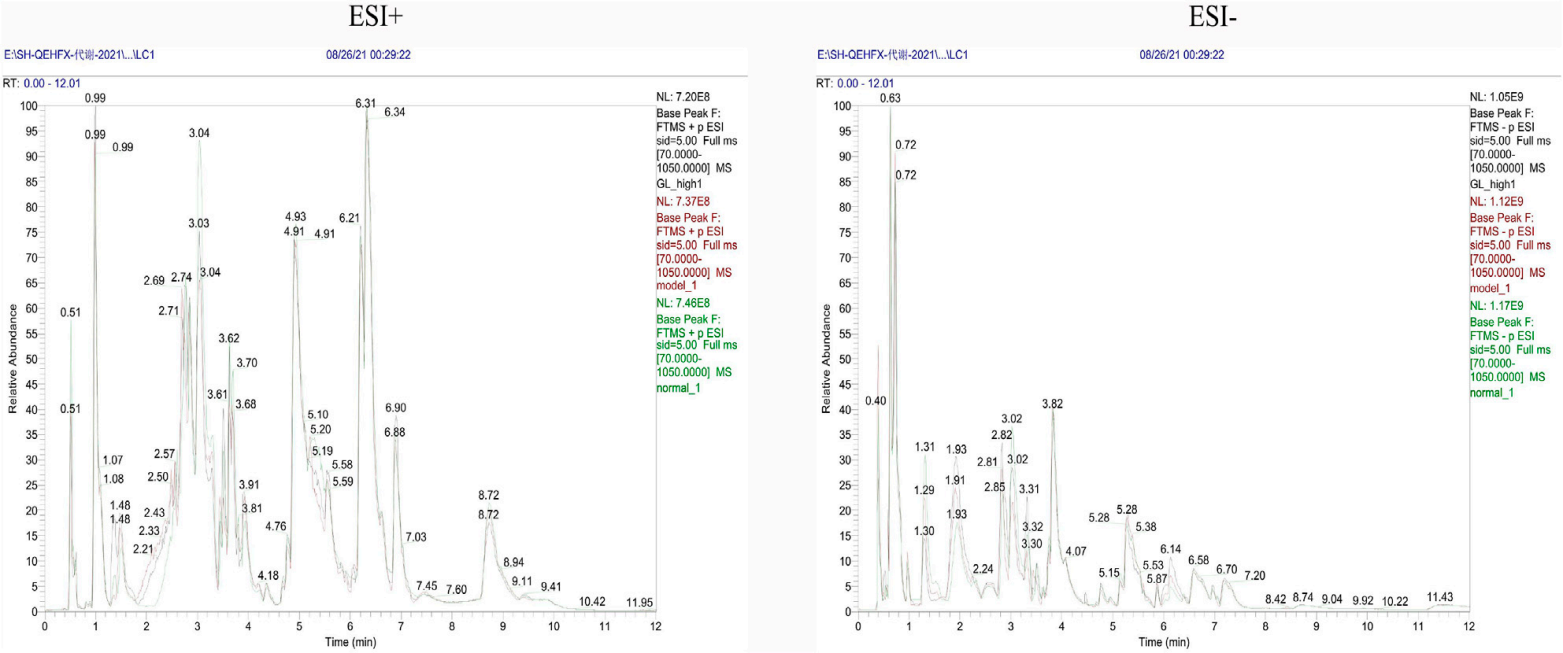
Representative BPC of rat liver tissue in positive and negative ion mode for each group.

Endogenous metabolites were detected in positive and negative ionization modes by UHPLC-Q Exactiv and the Base Peak Chromatogram (BPC) obtained for each group (Figure 3).

**FIGURE 4:**
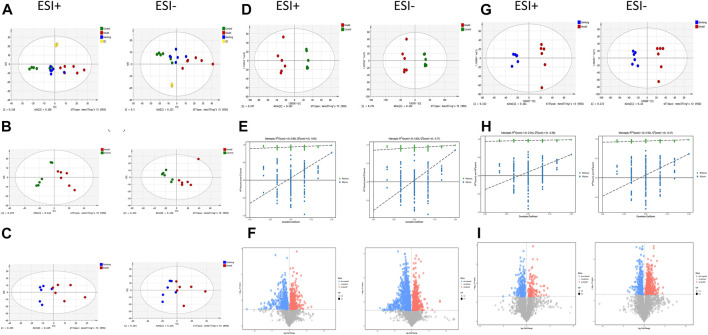
Metabolomic analysis of liver tissues of rats with liver fibrosis by Ganlong capsules. **(A)** Plots of liver tissue PCA scores between positive and negative ion normal control group, Ganlong capsule group and model group rats. **(B)** PCA plots of liver tissues between model group vs. normal control group and **(C)** Ganlong capsule group vs. model group rats in positive and negative ion mode. **(D–F)** Plots of OPLS-DA scores, 200th response ranking test plots and volcano plots between normal control and model groups in positive and negative ion mode. **(G–I)** Plots of OPLS-DA scores, 200 response sorting test plots and volcanoes between the model and Ganlong capsule groups in positive and negative ion mode. Control group, model group and Ganlong capsule treatment group (*n* = 6).

PCA showed tight aggregation in both positive and negative ion modes for quality control samples (QC), indicating good system stability and reproducibility (Figure 4A). R2X values were all greater than 0.5. Significant separation between the fibrosis model and control groups was seen in both positive and negative ion patterns, indicating significant differences in metabolic profile. Values for the group treated with *ganlong capsules* showed a trend away from the model group tending towards the normal control group. Two-by-two analysis revealed that the control and model groups were completely separated in the positive and negative ion mode (Figure 4B) and that the *ganlong capsules* administration group was separated from the model group (Figure 4C).

OPLS-DA analysis of metabolic profiles showed the separations of both the normal control and *ganlong capsules* group from the fibrosis model group in positive and negative ion mode (Figures 4D, G). OPLS-DA parameters gave R2Y values greater than 0.9 and Q2 values greater than 0.5 with positive slopes for R2Y and Q2 and negative intercepts for Q2, indicating no “overfitting” and good validity and reliability (Figures 4E, H). VIP, P, and FC values were visualized by volcano plot to enable screening for differential metabolites (Figures 4F, I).

Intraperitoneal injection of CCl_4_ led to significant differences in liver metabolic profiles between the model and control groups, indicating the successful generation of the liver fibrosis model at the metabolic level. Separation of the *ganlong capsules* and model groups indicated alterations of liver metabolic profiles resulting from treatment.

**FIGURE 5:**
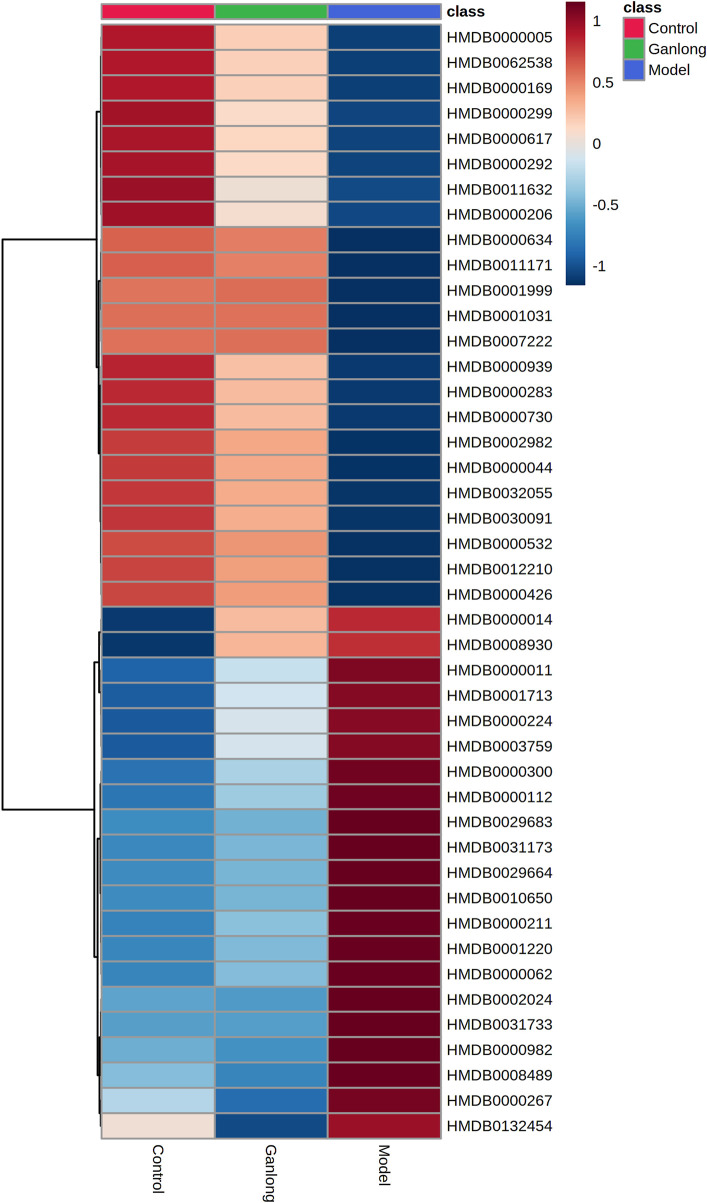
Heat map of potential biomarker expression in liver tissue of rats in each group. Control group, model group and Ganlong capsule treatment group (*n*=6). Model group compared with normal control group, ^*^ *p* < 0.05, ^**^ *p* < 0.01; Ganlong capsule group compared with model group, ^#^ *p* < 0.05, ^##^ *p* < 0.01.

A total of 43 differential metabolites were identified with a VIP >1 and *p* < 0.05 (fold change (FC) > 1.2 or <0.8) between model vs. control groups or *Ganlong capsules* vs. model groups (Table 1). Clustering analysis was performed *via* the MetaboAnalyst 5.0 website (Figure 5).

**TABLE 1:** Summary of the differential metabolites identified in the liver tissue of three Groups.

No	metabolites	HMDB ID	t_R_/min	m/z	Model/Control			Ganlong/Model		
					VIP	FC	trend	VIP	FC	trend
ESI+										
1	DG (18:1 (9Z)/18:4 (6Z, 9Z, 12Z, 15Z)/0:0)	HMDB0007222	0.51	615.4996	1.33	0.58	↓^*^	1.65	1.73	↑^a^
2	5a-Pregnane-3, 20-dione	HMDB0003759	0.54	317.2479	1.21	2.14	↑^*^	1.51	0.55	↓^a^
3	4- (4-Methyl-3-pentenyl) -3-cyclohexene-1-carboxaldehyde	HMDB0031733	0.61	193.1589	1.51	1.61	↑^*^	1.72	0.62	↓^a^
4	PG (18:2 (9Z, 12Z)/18:2 (9Z, 12Z))	HMDB0010650	0.64	771.5169	1.70	1.66	↑^**^	2.22	0.63	↓^b^
5	6-Hydroxy-1H-indole-3-acetamide	HMDB0031173	0.70	191.0814	1.60	2.54	↑^*^	1.93	0.43	↓^a^
6	Vanilloside	HMDB0029664	0.82	315.1089	1.46	2.47	↑^*^	1.75	0.41	↓^a^
7	PC (20:4 (8Z, 11Z, 14Z, 17Z)/P-18:0)	HMDB0008489	2.48	794.6074	1.77	3.08	↑^*^	1.94	0.32	↓^a^
8	PE (16:0/18:3 (9Z, 12Z, 15Z) i)	HMDB0008930	2.95	714.5068	1.63	3.91	↑^**^	1.71	0.65	↓^a^
9	5-Methylcytidine	HMDB0000982	4.72	258.1083	1.50	1.51	↑^**^	2.05	0.63	↓^b^
10	Xanthosine	HMDB0000299	5.37	285.0835	1.74	0.26	↓^**^	1.59	2.08	↑^a^
11	Xanthine	HMDB0000292	5.37	153.0407	1.71	0.27	↓^**^	1.58	2.03	↑^a^
12	N-Acetylhistidine	HMDB0032055	5.45	198.0976	1.72	0.22	↓^*^	1.91	3.35	↑^a^
13	beta-D-Glucosamine	HMDB0030091	5.45	180.0869	1.64	0.18	↓^*^	1.82	3.24	↑^b^
14	L-Carnitine	HMDB0000062	6.35	162.1124	1.51	1.36	↑^*^	1.85	0.76	↓^a^
15	N6-Acetyl-L-lysine	HMDB0000206	6.96	189.1233	1.67	0.56	↓^**^	1.51	1.35	↑^a^
16	gamma-Glutamylleucine	HMDB0011171	7.11	261.1448	1.48	0.47	↓^**^	1.68	2.01	↑^b^
ESI-										
17	Ascorbic acid	HMDB0000044	0.58	175.0243	1.60	0.31	↓^**^	1.65	2.76	↑^a^
18	Eicosapentaenoic acid	HMDB0001999	0.64	301.2170	1.15	0.48	↓^*^	1.47	2.11	↑^a^
19	Acetylglycine	HMDB0000532	0.80	116.0343	1.45	0.34	↓^**^	1.62	2.79	↑^a^
20	2-Hydroxy-6-pentadecylbenzoic acid	HMDB0029683	0.82	347.2596	1.76	6.35	↑^*^	1.75	0.28	↓^a^
21	m-Coumaric acid	HMDB0001713	0.95	163.0395	1.67	1.73	↑^**^	1.83	0.72	↓^a^
22	Imidazoleacetic acid	HMDB0002024	1.12	125.0346	1.41	2.98	↑^*^	1.73	0.35	↓^a^
23	Citraconic acid	HMDB0000634	1.23	129.0183	1.22	0.48	↓^*^	1.74	1.92	↑^b^
24	Uracil	HMDB0000300	1.30	111.0188	1.53	2.98	↑^**^	1.78	0.45	↓^a^
25	Deoxyribose 5-phosphate	HMDB0001031	1.34	213.0166	1.30	0.62	↓^*^	1.56	1.64	↑^a^
26	Dihydrolipoate	HMDB0012210	1.40	207.0504	1.29	0.62	↓^*^	1.35	1.46	↑^a^
27	Prostaglandin B1	HMDB0002982	1.67	335.2236	1.37	0.26	↓^**^	1.58	2.60	↑^a^
28	Prostaglandin E2	HMDB0001220	1.82	351.2175	1.36	3.08	↑^*^	1.68	0.36	↓^a^
29	D-Ribose	HMDB0000283	2.26	149.0448	1.45	0.37	↓^**^	1.42	1.93	↑^a^
30	2-Furoic acid	HMDB0000617	2.60	111.0078	1.65	0.30	↓^**^	1.58	2.06	↑^a^
31	Deoxycytidine	HMDB0000014	3.76	226.0831	1.17	4.46	↑^**^	2.13	0.49	↓^b^
32	Isobutyrylglycine	HMDB0000730	3.77	144.0657	1.54	0.36	↓^*^	1.85	1.99	↑^a^
33	(R) -3-Hydroxybutyric acid	HMDB0000011	3.96	103.0390	1.26	1.61	↑^*^	1.62	0.70	↓^a^
34	Pyroglutamic acid	HMDB0000267	5.16	128.0343	1.12	1.30	↑^*^	1.51	0.70	↓^a^
35	L-Iditol	HMDB0011632	5.25	181.0712	1.62	0.19	↓^*^	1.55	2.26	↑^a^
36	Fructose-1P	HMDB0062538	5.39	179.0555	1.60	0.29	↓^**^	1.64	2.11	↑^b^
37	2-Ketobutyric acid	HMDB0000005	5.41	101.0235	1.70	0.32	↓^**^	1.85	2.00	↑^b^
38	gamma-Aminobutyric acid	HMDB0000112	5.79	102.0550	1.39	2.08	↑^*^	1.52	0.56	↓^a^
39	Citramalic acid	HMDB0000426	6.16	147.0292	1.39	0.64	↓^*^	1.48	1.45	↑^a^
40	D-Mannose	HMDB0000169	6.49	179.0555	1.65	0.37	↓^**^	1.56	1.93	↑^a^
41	myo-Inositol	HMDB0000211	6.92	179.0555	1.63	1.55	↑^**^	1.66	0.70	↓^a^
42	S-Adenosylhomocysteine	HMDB0000939	7.07	383.1152	1.61	0.37	↓^**^	1.75	1.95	↑^b^
43	O-Phosphoethanolamine	HMDB0000224	8.54	140.0108	1.77	2.59	↑^*^	1.85	0.57	↓^a^

Control group, model group and Ganlong capsule treatment group (*n* = 6). Model group compared with normal control group, *p < 0.05, **p < 0.01; Ganlong capsule group compared with model group.

a *p* < 0.05.

b *p* < 0.01.

Levels of phosphatidylethanolamine [PE (16:0/18:3 (9Z, 12Z, 15Z], phosphatidylcholine [PC (20:4 (8Z, 11Z, 14Z, 17Z)/P-18:0)], ethanolamine phosphate (O-Phosphoethanolamine) (R) -3-hydroxybutyric acid (R) -3-Hydroxybutyric acid), gamma-Aminobutyric acid (gamma-Aminobutyric acid) and 20 other metabolites were significantly elevated in fibrotic liver tissue compared with controls (*p* < 0.05) and 23 metabolites, including 2-ketobutyric acid (2-Ketobutyric acid), were significantly decreased (*p* < 0.05). *Ganlong capsule* administration produced a significant trend of regression towards control values (*p* < 0.05).

The pathways of glycerophospholipid metabolism (*p* = 0.042, impact = 0.223) and butanoate metabolism (*p* = 0.041, impact = 0.032) were analyzed on the basis of values of *p* < 0.05 (Table 2).

**TABLE 2:** Analysis of metabolic pathways in liver tissues based on potential biomarkers

No	Pathway Name	Match Status	Raw *p*	-log (*p*)	Impact
1	Butanoate metabolism	1/15	0.041	1.387	0.032
2	Glycerophospholipid metabolism	3/36	0.042	1.376	0.223
3	Pentose phosphate pathway	2/22	0.082	1.086	0.092
4	Synthesis and degradation of ketone bodies	1/5	0.105	0.978	0.000
5	Linoleic acid metabolism	1/5	0.105	0.978	0.000
6	Cysteine and methionine metabolism	2/33	0.162	0.791	0.086
7	Ascorbate and aldarate metabolism	1/8	0.163	0.788	0.000
8	Valine, leucine and isoleucine biosynthesis	1/8	0.163	0.788	0.000
9	Arachidonic acid metabolism	2/36	0.186	0.731	0.000
10	Pyrimidine metabolism	2/39	0.210	0.678	0.079
11	alpha-Linolenic acid metabolism	1/13	0.251	0.600	0.000
12	Glycosylphosphatidylinositol (GPI) -anchor biosynthesis	1/14	0.268	0.572	0.004
13	Histidine metabolism	1/16	0.300	0.523	0.000
14	Pantothenate and CoA biosynthesis	1/19	0.346	0.462	0.000
15	beta-Alanine metabolism	1/21	0.374	0.427	0.000
16	Sphingolipid metabolism	1/21	0.374	0.427	0.014
17	Propanoate metabolism	1/23	0.402	0.396	0.041
18	Purine metabolism	2/65	0.422	0.375	0.012
19	Galactose metabolism	1/27	0.453	0.344	0.000
20	Glutathione metabolism	1/28	0.466	0.332	0.007
21	Phosphatidylinositol signaling system	1/28	0.466	0.332	0.037
22	Alanine, aspartate and glutamate metabolism	1/28	0.466	0.332	0.087
23	Inositol phosphate metabolism	1/30	0.489	0.311	0.129
24	Glycine, serine and threonine metabolism	1/33	0.523	0.282	0.000
25	Biosynthesis of unsaturated fatty acids	1/36	0.554	0.256	0.000
26	Arginine and proline metabolism	1/38	0.574	0.241	0.024
27	Steroid hormone biosynthesis	1/85	0.856	0.067	0.015

Control group, model group and Ganlong capsule treatment group (*n* = 6) Model group compared with normal control group, *p < 0.05, **p < 0.01. Ganlong capsule group compared with model group, ^#^ *p* < 0.05, ^##^ *p* < 0.01.

### Multivariate analysis of serum metabolomics

**FIGURE 6:**
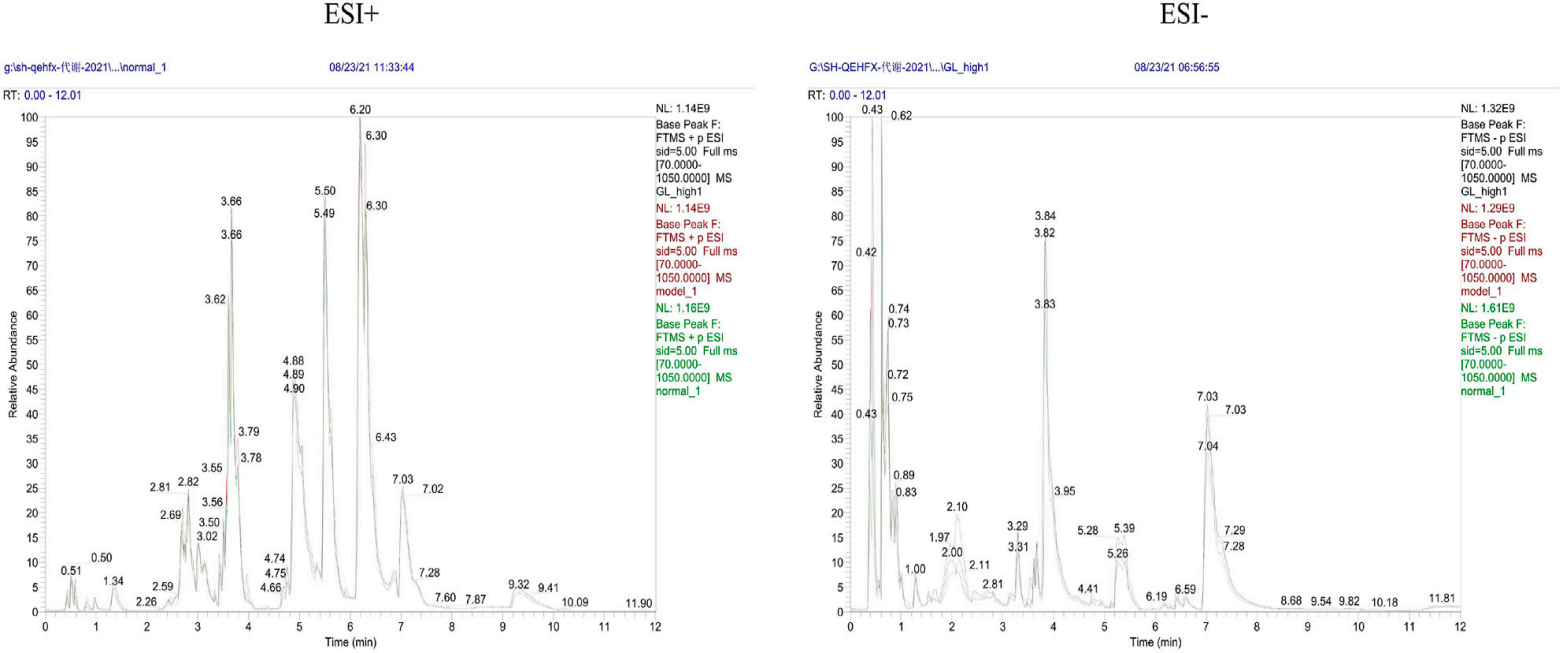
Representative BPC of rat Serum in positive and negative ion mode for each group.

Metabolic characteristics of endogenous serum metabolites were analyzed by UHPLC-Q Exactive system and serum BPC plots obtained in positive and negative ion mode (Figure 6).

**FIGURE 7:**
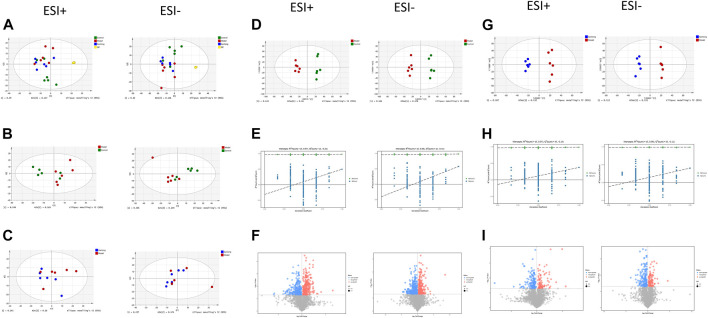
Serum metabolomic analysis of Ganlong capsule on liver fibrosis in rats. **(A)** Plots of liver tissue PCA scores between positive and negative ion normal control group, ganlong capsule group and model group rats. **(B)** PCA plots of liver tissues between model group vs. normal control group and **(C)** Ganlong capsule group vs. model group rats in positive and negative ion mode. **(D–F)** Plots of OPLS-DA scores, 200th response ranking test plots and volcano plots between normal control and model groups in positive and negative ion mode. **(G–I)** Plots of OPLS-DA scores, 200 response sorting test plots and volcanoes between the model and ganlong capsule groups in positive and negative ion mode. Control group, model group and ganlong capsule treated group (*n* = 6).

Unsupervised PCA analysis showed aggregation of QC samples in positive and negative ion modes, indicating good stability and reliability. Some separation of the control, model and *Ganlong capsule* groups was shown but was incomplete, perhaps due to noise interference (Figure 7A). PCA maps also showed incomplete separation of the model *versus* control groups and the *Ganlong capsule versus* model groups in the positive and negative ionization mode (Figures 7B, C).

Supervised OPLS-DA modeling analysis was implemented to eliminate noise and showed the control group to be completely separated from the model group and the *Ganlong capsule* group to be separated from the model group under the positive and negativeion model (Figures 7D, G). R2Y values were greater than 0.9 but Q2 values mostly less than 0.5, indicating excellent explanatory but poor predictive ability of the models. Response ranking tests gave positive R2Y and Q2 slopes and a negative Q2 intercept, excluding “overfitting” and confirming validity and reliability (Figures 7E, H). Red and blue dots represent differential metabolites meeting the conditions of VIP >1, P < 0.05 and FC > 1.2 or FC < 0.8 in Figures 7F, I.

A total of 21 potential biomarkers were identified in rat serum as being either up- or downregulated (*p* < 0.05) by *Ganlong capsule* treatment. Serum PC (PC (20:2 (11Z, 14Z)/20:4 (5Z, 8Z, 11Z, 14Z)), PC (20:4 (8Z, 11Z, 14Z, 17Z)/P-18:0)), PE (PE (O-16:1 (1Z)/22:6 (4Z, 7Z, 10Z 13Z, 16Z, 19Z)) and thymidine (thymine) showed 17 significantly elevated metabolites (*p* < 0.05) and malonic acid four significantly lower metabolites in the model compared with control groups and restoration of values was shown for all in the *Ganlong capsule* group (*p* < 0.05; Table 3). Heat maps were drawn using the Metaboanalyst 5.0 website to visualize clustering of potential serum biomarkers.

**TABLE 3:** Summary of the differential metabolites identified in the serum of three Groups.

No	metabolites	HMDB ID	t_R_/min	m/z	Model/Control			Ganlong/Model		
					VIP	FC	trend	VIP	FC	trend
ESI+										
1	Dieporeticenin	HMDB0029792	0.51	573.4884	1.83	0.07	↓^a^	1.77	4.35	↑^c^
2	1H-Indole-3-carboxaldehyde	HMDB0029737	0.79	146.0599	1.90	0.76	↓^b^	2.13	1.27	↑^c^
3	Thymine	HMDB0000262	1.46	127.0503	2.14	1.72	↑^b^	2.34	0.72	↓^d^
4	PC (P-18:1 (11Z)/22:5 (4Z, 7Z, 10Z, 13Z, 16Z))	HMDB0011293	2.62	818.6078	2.29	3.05	↑^b^	2.15	0.55	↓^c^
5	PE (P-18:1 (11Z)/22:5 (4Z, 7Z, 10Z, 13Z, 16Z))	HMDB0011425	2.62	776.5596	2.04	1.96	↑^b^	2.06	0.64	↓^c^
6	PC (20:2 (11Z, 14Z)/20:4 (5Z, 8Z, 11Z, 14Z))	HMDB0008345	2.64	834.6009	1.69	1.37	↑^a^	2.19	0.70	↓^c^
7	PE (P-18:1 (9Z)/20:4 (5Z, 8Z, 11Z, 14Z))	HMDB0011451	2.65	750.5438	2.17	2.03	↑^b^	1.84	0.68	↓^c^
8	PE (O-16:1 (1Z)/22:6 (4Z, 7Z, 10Z, 13Z, 16Z, 19Z))	HMDB0005780	2.65	748.5291	2.37	1.89	↑^b^	2.06	0.70	↓^c^
9	PC (20:4 (8Z, 11Z, 14Z, 17Z)/P-18:0)	HMDB0008489	2.66	794.6076	2.34	1.89	↑^b^	1.91	0.70	↓^c^
10	Vaccenyl carnitine	HMDB0006351	3.31	426.3584	2.34	1.90	↑^b^	1.96	0.85	↓^c^
11	SM (d16:1/24:1 (15Z))	HMDB0011694	3.36	785.6528	1.70	1.34	↑^a^	1.97	0.75	↓^c^
12	cis-5-Tetradecenoylcarnitine	HMDB0002014	3.43	370.2948	2.14	1.73	↑^b^	2.06	0.77	↓^c^
13	Dodecanoylcarnitine	HMDB0002250	3.51	344.2793	1.93	1.68	↑^b^	1.88	0.72	↓^c^
14	Trimethylamine N-oxide	HMDB0000925	5.88	76.0762	1.94	2.97	↑^b^	2.53	0.27	↓^d^
15	Lysyl-Valine	HMDB0028964	9.65	246.1814	2.16	1.60	↑^b^	2.03	0.71	↓^c^
ESI-										
16	Hexylresorcinol	HMDB0032567	0.81	193.1229	1.80	1.46	↑^b^	1.76	0.82	↓^c^
17	2-Furoic acid	HMDB0000617	0.82	111.0078	1.52	0.51	↓^a^	2.07	1.77	↓^c^
18	Maslinic acid	HMDB0002392	0.93	471.3488	2.07	4.74	↑^a^	2.46	0.28	↓^c^
19	Thymidine	HMDB0000273	1.36	241.0833	1.84	1.39	↑^b^	2.05	0.83	↓^c^
20	Malonic acid	HMDB0000691	1.42	103.0027	1.23	0.56	↓^a^	1.80	1.70	↓^c^
21	p-Cresol	HMDB0001858	3.11	107.0493	1.61	2.31	↑^a^	1.72	0.48	↓^c^

Control group, model group and Ganlong capsule treatment group (*n* = 6). Model group compared with normal control group.

a *p* < 0.05.

b *p* < 0.01. Ganlong capsule group compared with model group.

c *p* < 0.05.

d *p* < 0.01.

**FIGURE 8:**
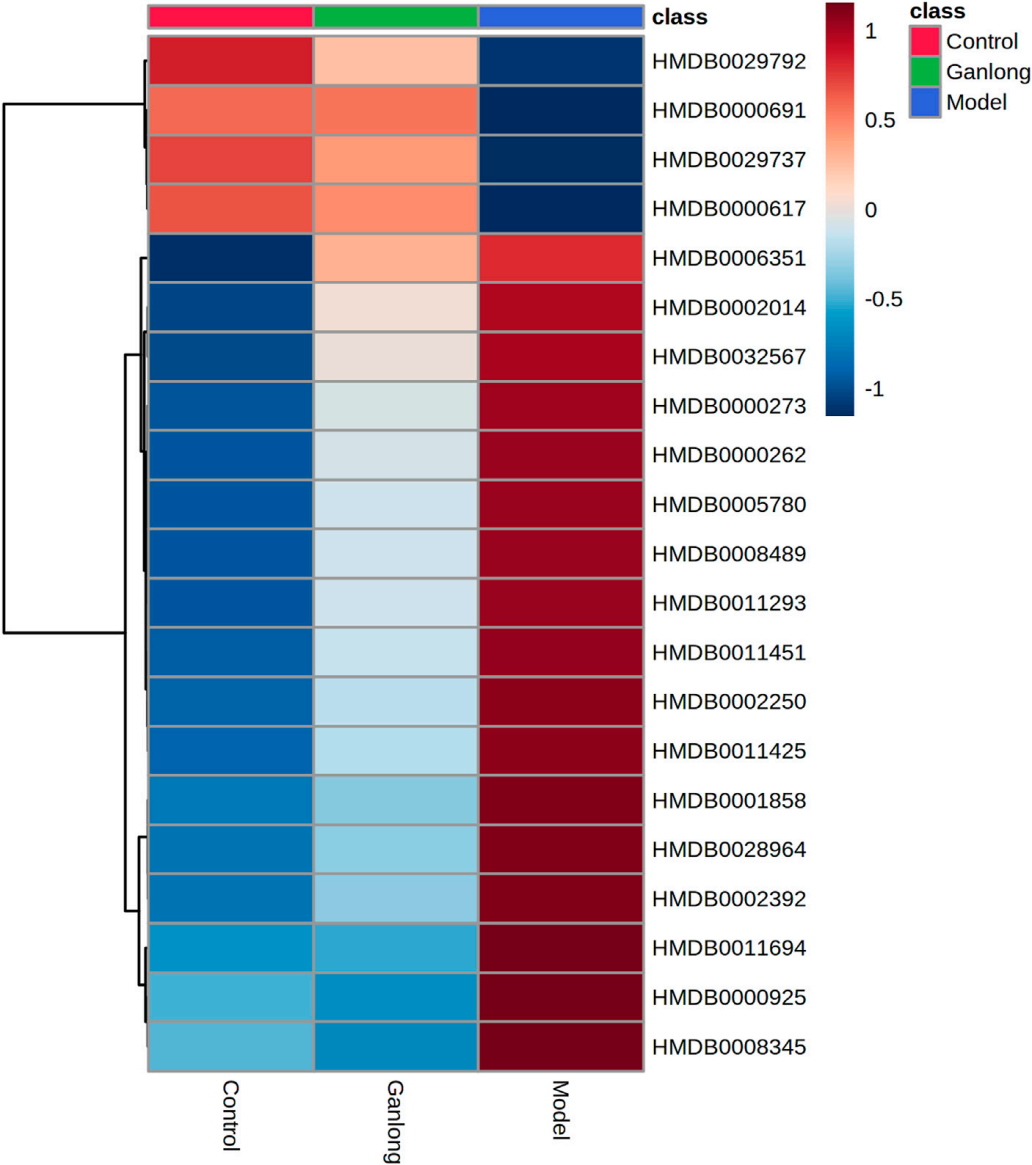
Heat map of potential biomarker expression in serum of rats in each group. Control group, model group and Ganlong capsule treatment group (*n*=6). Model group compared with normal control group, ^*^ *p* < 0.05, ^**^ *p* < 0.01. Ganlong capsule group compared with model group, ^#^ *p* < 0.05, ^##^ *p* < 0.01.

Pathways of glycerophospholipid metabolism (*p* = 0.021, impact = 0.199), pyrimidine metabolism (*p* = 0.024, impact = 0.097) and linoleic acid metabolism (*p* = 0.032, impact = 0.00) were analyzed since metabolites had values of *p* < 0.05 (Figure 8 and Table 4).

**TABLE 4:** Analysis of metabolic pathways in serum based on potential biomarkers

No	Pathway Name	Match Status	Raw *p*	-log (*p*)	Impact
1	Glycerophospholipid metabolism	2/36	0.021	1.678	0.199
2	Pyrimidine metabolism	2/39	0.024	1.612	0.097
3	Linoleic acid metabolism	1/5	0.032	1.496	0.000
4	alpha-Linolenic acid metabolism	1/13	0.081	1.092	0.000
5	Glycosylphosphatidylinositol (GPI) -anchor biosynthesis	1/14	0.087	1.061	0.004
6	Arachidonic acid metabolism	1/36	0.210	0.678	0.000

Control group, model group and Ganlong capsule treatment group (*n* = 6). Model group compared with normal control group, *p < 0.05, **p < 0.01. Ganlong capsule group compared with model group, ^#^ *P* < 0.05, ^##^ *P* < 0.01.

### Multivariate analysis of urine metabolomics

**FIGURE 9:**
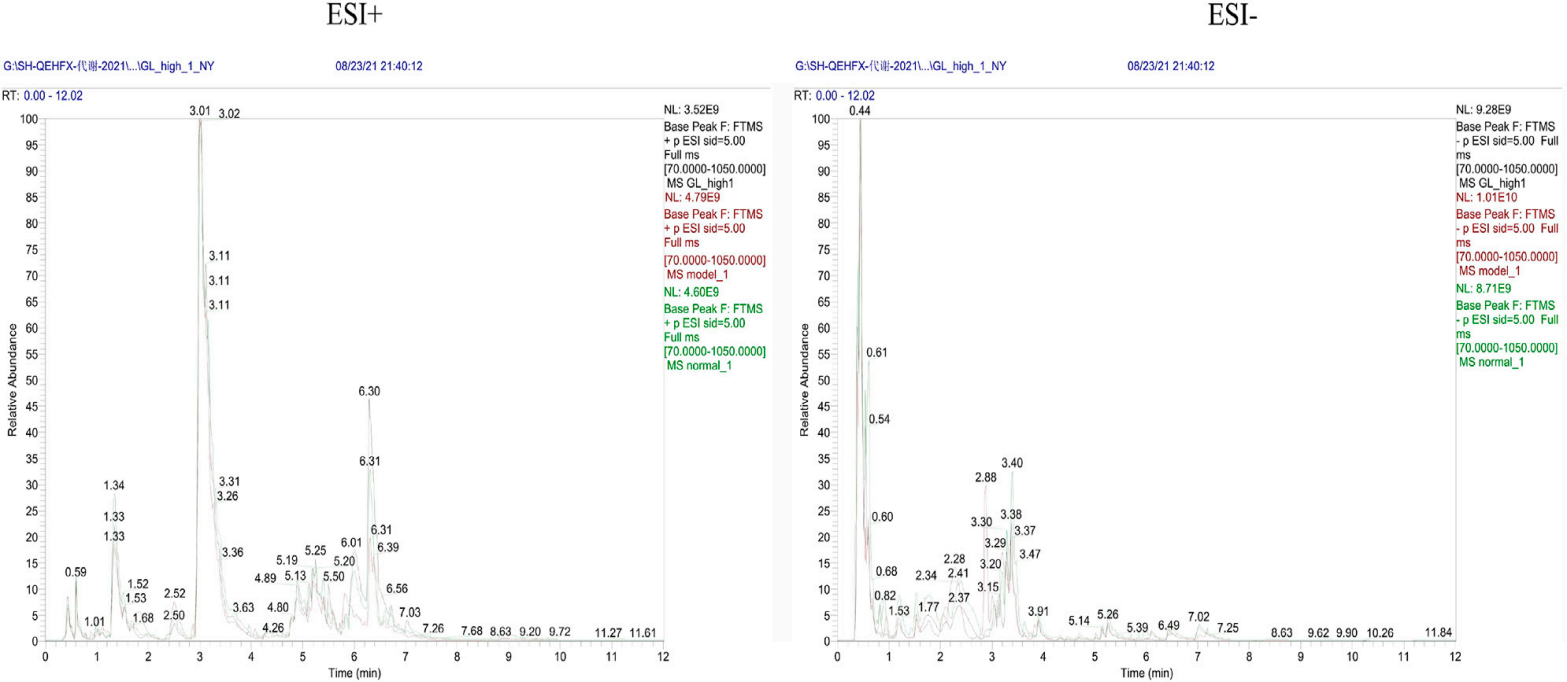
Representative BPC graph of rat urine in positive and negative ion mode for each group.

Metabolic characteristics of endogenous urine metabolites were analyzed by UHPLC-Q Exactive system and serum BPC plots obtained in positive and negative ion mode (Figure 9).

**FIGURE 10:**
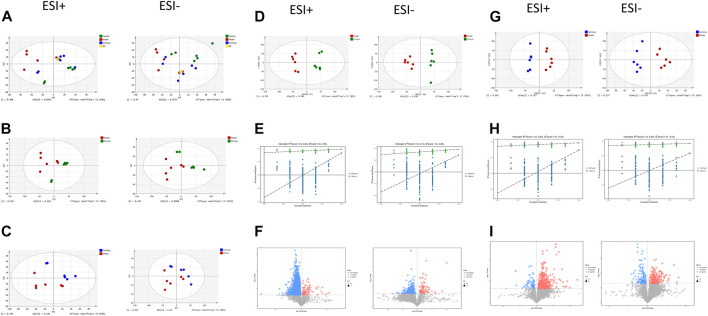
Urinary metabolomic analysis of ganlong capsule on liver fibrosis in rats. **(A)** Plots of liver tissue PCA scores between positive and negative ion normal control group, ganlong capsule group and model group rats. **(B)** PCA plots of liver tissues between model group vs. normal control group and **(C)** Ganlong capsule group vs. model group rats in positive and negative ion mode. **(D–F)** Plots of OPLS-DA scores, 200th response ranking test plots and volcano plots between normal control and model groups in positive and negative ion mode. **(G–I)** Plots of OPLS-DA scores, 200 response sorting test plots and volcanoes between the model and ganlong capsule groups in positive and negative ion mode. Control group, model group and ganlong capsule treated group (*n* = 6).

Unsupervised PCA of QC samples produced aggregated results showing good reliability. However, the separation of samples from the three groups was not clear in positive and negative ion modes (Figure 10A). PCA plots of model *versus* control (14 B) and *Ganlong capsule versus* model (14C) were separate in positive and negative ion mode with no outlier points.

OPLS-DA analysis was performed and validated by 200 times response ranking test. A volcano plot was constructed to identify potential biomarkers.

OPLS-DA results showed separation of controls from model and of *Ganlong capsules* from model samples in positive and negative ion mode. R2Y values were all greater than 0.9 and Q2 values all greater than 0.5 (Figures 10D, G). The 200 response ranking tests showed R2Y and Q2 values to be consistent with the model test parameters and “overfitting” to be absent (Figures 10F, H). Dot size corresponds to VIP value in the volcano plot with metabolites arranged in a V-shape, higher *p*-values at the bottom and smaller *p*-values at the top.

A total of 20 potential biomarkers which had been up- or downregulated by *Ganlong capsules* (*p* < 0.05) were identified in the urine of rats with liver fibrosis. Ginkgolide B levels were higher in urine from the model group (*p* < 0.05) and decreased after *Ganlong capsule* treatment (*p* < 0.05). Levels of 19 metabolites of choline (Choline), betaine aldehyde (Betaine), and L-leucine (L-Leucine) were lower in fibrotic urine (*p* < 0.05) and increased after *Ganlong capsule* treatment (*p* < 0.05) (Table 5). Heat maps of potential biomarker clusters were drawn *via* the Metaboanalyst 5.0 website.

**TABLE 5:** Summary of the differential metabolites identified in the urine of three Groups.

No	metabolites	HMDB ID	tR/sec	m/z	Model/Control			Ganlong/Model		
					VIP	FC	trend	VIP	FC	trend
ESI+										
1	Na, Na-Dimethylhistamine	HMDB0033438	0.29	140.1181	1.34	0.64	↓^*^	1.45	1.54	↑^a^
2	Adrenochrome	HMDB0012884	0.42	180.0655	1.11	0.32	↓^*^	1.70	3.13	↑^b^
3	2 (N) -Methyl-norsalsolinol	HMDB0001189	0.48	180.1018	1.38	0.29	↓^*^	1.96	4.63	↑^a^
4	Vanillylamine	HMDB0012309	0.51	154.0862	1.01	0.38	↓^*^	1.61	2.70	↑^a^
5	Meconine	HMDB0032652	0.59	195.0691	1.45	0.38	↓^**^	1.60	2.26	↑^a^
6	Homovanillin	HMDB0005175	0.65	167.0700	1.30	0.27	↓^*^	1.15	2.19	↑^a^
7	3-Methylpyrrolo [1, 2-a]pyrazine	HMDB0033172	2.32	134.0712	1.19	0.29	↓^*^	1.99	8.58	↑^a^
8	beta-D-Glucosamine	HMDB0030091	2.42	180.0877	1.37	0.30	↓^**^	1.36	2.49	↑^a^
9	Isopentyl beta-D-glucoside	HMDB0034750	2.55	251.1500	1.48	0.28	↓^*^	1.84	4.61	↑^a^
10	2-Methylbutyroylcarnitine	HMDB0000378	4.45	246.1700	1.23	0.29	↓^*^	1.82	4.15	↑^b^
11	4-Trimethylammoniobutanoic acid	HMDB0001161	4.52	146.1175	1.15	0.47	↓^*^	1.73	2.35	↑^a^
12	Alanyl-Proline	HMDB0028695	4.74	187.1077	1.22	0.43	↓^*^	1.96	4.40	↑^b^
13	Choline	HMDB0000097	5.14	104.1072	1.20	0.44	↓^**^	1.38	1.83	↑^a^
14	Naphthalene epoxide	HMDB0006215	5.27	143.0484	1.33	0.35	↓^**^	1.32	1.83	↑^a^
15	2-Acetylthiazole	HMDB0032964	5.30	128.0178	1.39	0.31	↓^**^	1.53	2.09	↑^a^
16	Hydroxyprolyl-Leucine	HMDB0028867	5.96	245.1606	1.65	0.20	↓^**^	1.70	2.52	↑^a^
17	L-Leucine	HMDB0000687	6.05	132.1020	1.49	0.28	↓^**^	1.76	3.29	↑^a^
18	Betaine aldehyde	HMDB0001252	6.30	102.0917	1.39	0.41	↓^**^	1.83	2.98	↑^a^
19	Lauroyl diethanolamide	HMDB0032358	9.86	288.2537	1.09	0.66	↓^*^	1.52	1.53	↑^a^
ESI-										
20	Ginkgolide B	HMDB0036861	6.01	423.1265	1.15	4.15	↑^*^	1.63	0.25	↓^a^

Control group, model group and Ganlong capsule treatment group (*n* = 6). Model group compared with normal control group, *p < 0.05, **p < 0.01. Ganlong capsule group compared with model group.

a *p* < 0.05.

b *p* < 0.01.

**FIGURE 11:**
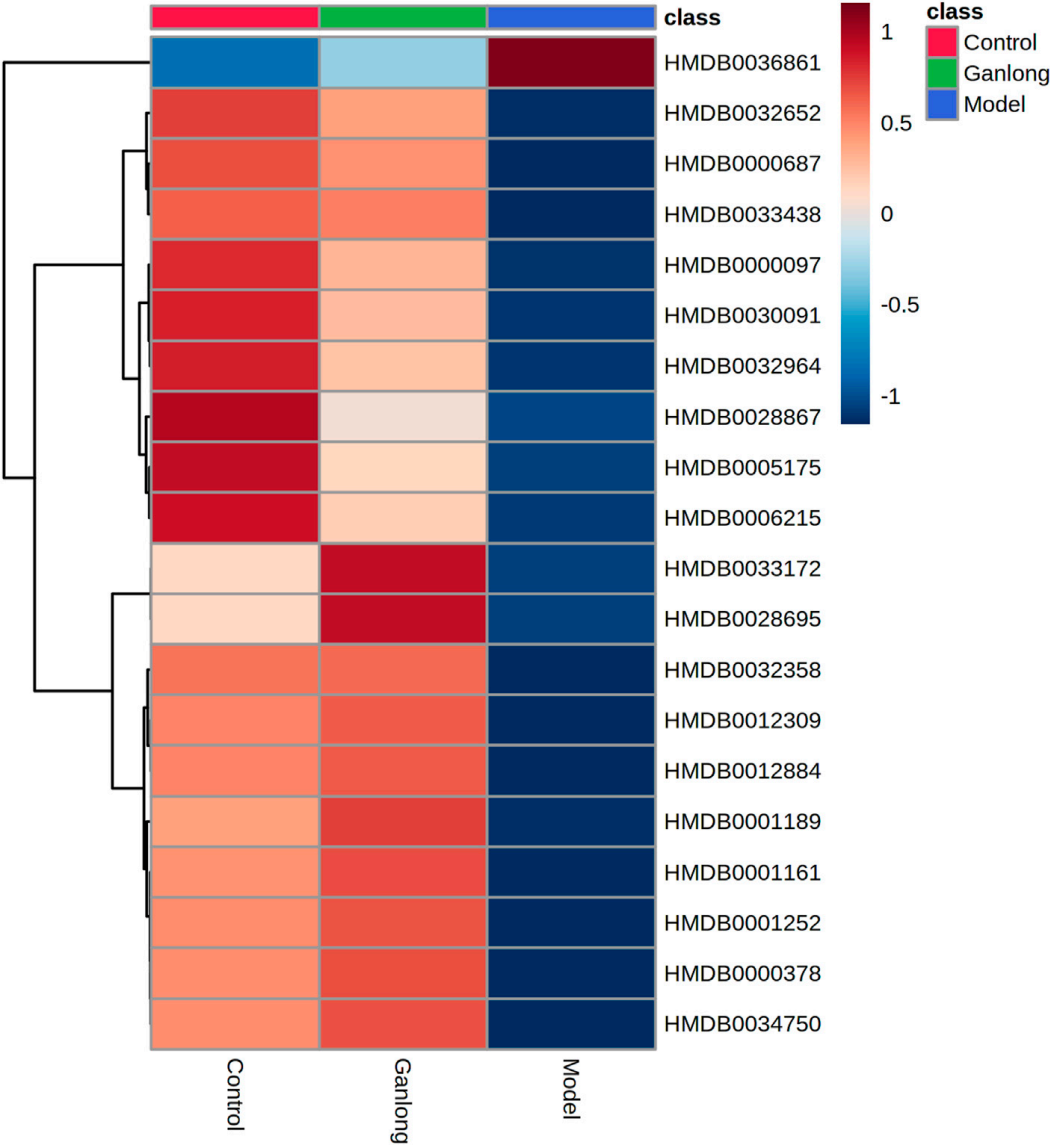
Heat map of potential biomarker expression in urine of rats in each group. Control group, model group and Ganlong capsule treatment group (*n*=6). Model group compared with normal control group, ^*^ *p* < 0.05, ^**^ *p* < 0.01. Ganlong capsule group compared with model group, ^#^ *p* < 0.05, ^##^ *p* < 0.01.

The identification of differential metabolites allowed the selection of two pathways of interest: glycine, serine and threonine metabolism (*p* = 0.014, impact = 0.026) and valine, leucine and isoleucine biosynthesis (*p* = 0.046, impact = 0.000; Figure 11 and Table 6).

**TABLE 6:** Analysis of metabolic pathways in urine based on potential biomarkers

No	Pathway Name	Match Status	Raw *p*	-log (*p*)	Impact
1	Glycine, serine and threonine metabolism	2/33	0.014	1.841	0.026
2	Valine, leucine and isoleucine biosynthesis	1/8	0.046	1.341	0.000
3	Lysine degradation	1/25	0.136	0.865	0.005
4	Glycerophospholipid metabolism	1/36	0.191	0.719	0.026
5	Valine, leucine and isoleucine degradation	1/40	0.210	0.677	0.000
6	Tyrosine metabolism	1/42	0.220	0.658	0.006
7	Aminoacyl-tRNA biosynthesis	1/48	0.247	0.607	0.000
8	Metabolism of xenobiotics by cytochrome P450	1/68	0.333	0.478	0.010

Control group, model group and Ganlong capsule treatment group (*n* = 6). Model group compared with normal control group, *p < 0.05, **p < 0.01. Ganlong capsule group compared with model group, ^#^ *p* < 0.05, ^##^ *p* < 0.01.

## Discussion

The hepatotoxin, CCl_4_, is frequently used to generate models of liver fibrosis for the screening and mechanistic study of anti-liver fibrosis drugs (Xi Chen et al., 2021). CCl_4_ causes infiltration of inflammatory cells, liver damage and ultimately liver fibrosis (Dong et al., 2016).

The current study utilized CCl_4_ to induce liver fibrosis in male SD rats and pharmacodynamic and metabolomic analyses allowed identification of key metabolic pathways *via* enrichment of differential metabolites in liver tissue, blood and urine.

Fibrosis model rats had increased liver index, serum ALT and AST levels and levels of fibrosis markers, HA, LN, PC III and Col-IV. Hepatocyte degeneration, inflammatory infiltration, fibrous connective tissue proliferation, structural damage to liver lobules and the creation of fibrous sept were seen during pathological analyses, indicating successful generation of the fibrosis model. *Ganlong capsule* treatment reduced serum ALT and AST values, liver fibrosis quadruple g values and liver index and improved pathological changes. In conclusion, *Ganlong capsule* treatment ameliorated CC1_4_-induced liver fibrosis.

Metabolomic analyses indicated changes in glycerophospholipid, linoleic acid, butyric acid, pyrimidine and amino acid metabolism amongst control, model and *Ganlong capsule*-treated groups. These findings illustrate the impact of *Ganlong capsules* on the above metabolic pathways which may account for the therapeutic effect on fibrosis.

### Lipid metabolism

Glycerophospholipid metabolism in liver (*p* = 0.042, impact = 0.223) and serum samples (*p* = 0.021, impact = 0.199) and linoleic acid metabolism (*p* = 0.032, impact = 0.000) were aspects of lipid metabolism found to be affected in CCl_4_-induced liver fibrosis. Glycerophospholipids, such as PE and PC, are key components of biological membranes and their metabolism affects membrane fusion, endocytosis and transport. Disorders of glycerophospholipid metabolism are thought to adversely affect the liver (Xue et al., 2020). The essential omega-6 polyunsaturated fatty acid (PUFA), linoleic acid, is essential for growth and development, cell function, signal transduction and the immune response (Lv et al., 2020). CCl_4_ has been shown to damage hepatocyte membranes and its metabolism by hepatocyte cytochrome P450 enzymes may generate free radicals. The consequent lipid peroxidation damages cell and organelle membranes, allowing accumulation of PC and PE, leading to hepatic steatosis and aggravating liver fibrosis (Weber et al., 2003; Yumeng et al., 2021). Raised PC and PE levels are considered to be markers of liver fibrosis in the clinic (Huang et al., 2011). The identification of five differential metabolites of PE, PC and ethanolamine phosphate with elevated levels during the present work indicates the involvement of glycerophospholipid and linoleic acid metabolism in fibrosis. *Ganlong capsule* treatment reduced the levels of these metabolites. Thus, *Ganlong capsules* ameliorated CCl_4_-disrupted glycerophospholipid metabolism, in agreement with previous reports (Yongcan, 2022).

### Butyric acid metabolism

Altered butyric acid metabolism is central to CCl_4_-induced liver fibrosis with elevated levels of gamma-aminobutyric acid (GABA), an inhibitory neuro-signalling transmitter, being involved (Zhu et al., 2018). Liver fibrosis and cirrhosis are thought to be associated with an imbalance in amino acid metabolism that raises blood ammonia levels and triggers hepatic encephalopathy (Butterworth, 2019). The present study found two metabolites of butyric acid (R) -3-hydroxybutyric acid and γ-aminobutyric acid, to be significantly upregulated in the fibrosis model, a change which was reversed by *Ganlong capsule* treatment.

### Pyrimidine metabolism

Liver tissue has a regenerative capacity which depends on active pyrimidine anabolism. Furthermore, inhibition of pyrimidine synthesis prevents excessive cell proliferation and protects against liver fibrosis (Cunshuan, 2010). Thymidine levels were greatly enhanced in the liver fibrosis model of the current study and decreased following *Ganlong capsule* administration. CCl_4_-damaged liver tissue may have activated the self-protective mechanism of secreting ECM, increasing formation of collagen fibres. In contrast, *Ganlong capsules* reduced pyrimidine levels while alleviating abnormal proliferation of liver cells, reversing liver fibrosis. The levels of deoxycytidine and uracil were much higher in model rats than in controls, consistent with previous reports (Liu et al., 2013). *Ganlong capsules* reduced deoxycytidine and uracil levels to approximate control values. Thus, *Ganlong capsules* may exert anti-liver fibrosis effects by lowering pyrimidine levels and reducing excessive tissue proliferation.

### Amino acid metabolism

The present study showed glycine, serine and threonine metabolism and valine, leucine and isoleucine biosynthesis to be involved in CCl_4_-induced liver fibrosis. The liver is a key site for amino acid metabolism and damage to this organ is known to impact these metabolic pathways (Trefts et al., 2017).

Choline and betaine aldehyde, involved in glycine, serine and threonine metabolism and L-leucine, involved in valine, leucine and isoleucine biosynthesis, were metabolites identified during the present work as being central to fibrosis. Choline and betaine aldehyde were lower in fibrosis but restored by *Ganlong capsules*. This suggests that CCl_4_-induced liver fibrosis is associated with glycine, serine and threonine metabolism and *Ganlong capsules* exert hepatoprotective effects through an impact on these pathways. The branched-chain amino acid, L-leucine, was lower in fibrosis and restored by *Ganlong capsules*. Thus, *Ganlong capsules* may exert anti-fibrotic effects *via* influencing branched chain amino acid metabolism.

### Other metabolism

Liver damage, resulting from fibrosis or hepatitis, is characterized by an inflammatory state. Arachidonic acid is stored as a membrane phospholipid and converted to inflammation-inducing prostaglandins by cyclooxygenase to exacerbate inflammation (Stringer, et al., 2016). The current investigation found arachidonic acid metabolism to be disturbed by CCl_4_, producing high levels of prostaglandin E2 which was reduced by *Ganlong capsules*. This may be another route by which *Ganlong capsules* exert a hepatoprotective effect.

## Conclusion and future prospectives

In conclusion, *Ganlong capsules* have a protective effect against hepatic fibrosis. Metabolomic analysis of liver, serum and urine after treatment with the proprietary Chinese medicine, *Ganlong capsules,* in a rat model of CCl_4_-induced hepatic fibrosis was conducted. The hepatoprotective effect of *Ganlong capsules* was found to be related to regulation of butyric acid, glycerophospholipid, linoleic acid, pyrimidine, glycine, serine and threonine metabolism and to biosynthesis of valine, leucine and isoleucine.

## Data Availability

The original contributions presented in the study are included in the article/Supplementary Material, further inquiries can be directed to the corresponding author.
