# Corrigendum: Metabonomic analysis of the anti-hepatic fibrosis effect of *Ganlong capsules*

**DOI:** 10.3389/fphar.2023.1231544

**Published:** 2023-07-07

**Authors:** ChangLing Lv, YinRui Li, Ling Ou, Jie Zhou, Fang Peng, DingYu Wu

**Affiliations:** ^1^ College of Pharmacy, Dali University, Dali, China; ^2^ Yunnan Provincial Key Laboratory of Entomological Biopharmaceutical R&D, Dali, China; ^3^ Department of Pharmacy, Mengzi People’s Hospital, Mengzi, China

**Keywords:** Ganlong capsule, hepatic fibrosis, CCl_4_, metabolomic analysis, LC-MS

In the published article, there was an error in Figures 3, 6, 9 as published. The reason for this is a mistake was made in the order of BPC pictures of different tissue samples. The corrected **Figures 3, 6 and 9** and their captions appear below.

**FIGURE 3:**
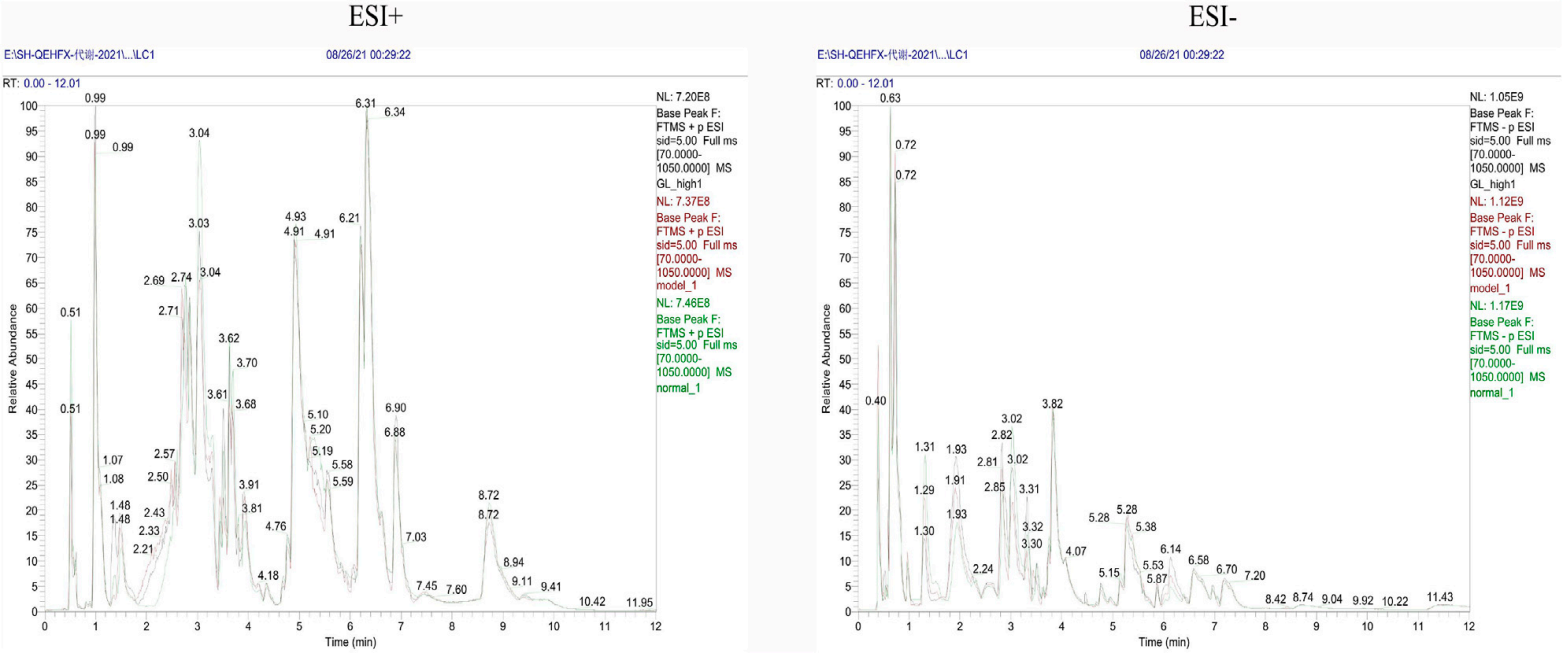
Representative BPC of rat liver tissue in positive and negative ion mode for each group.

**FIGURE 6:**
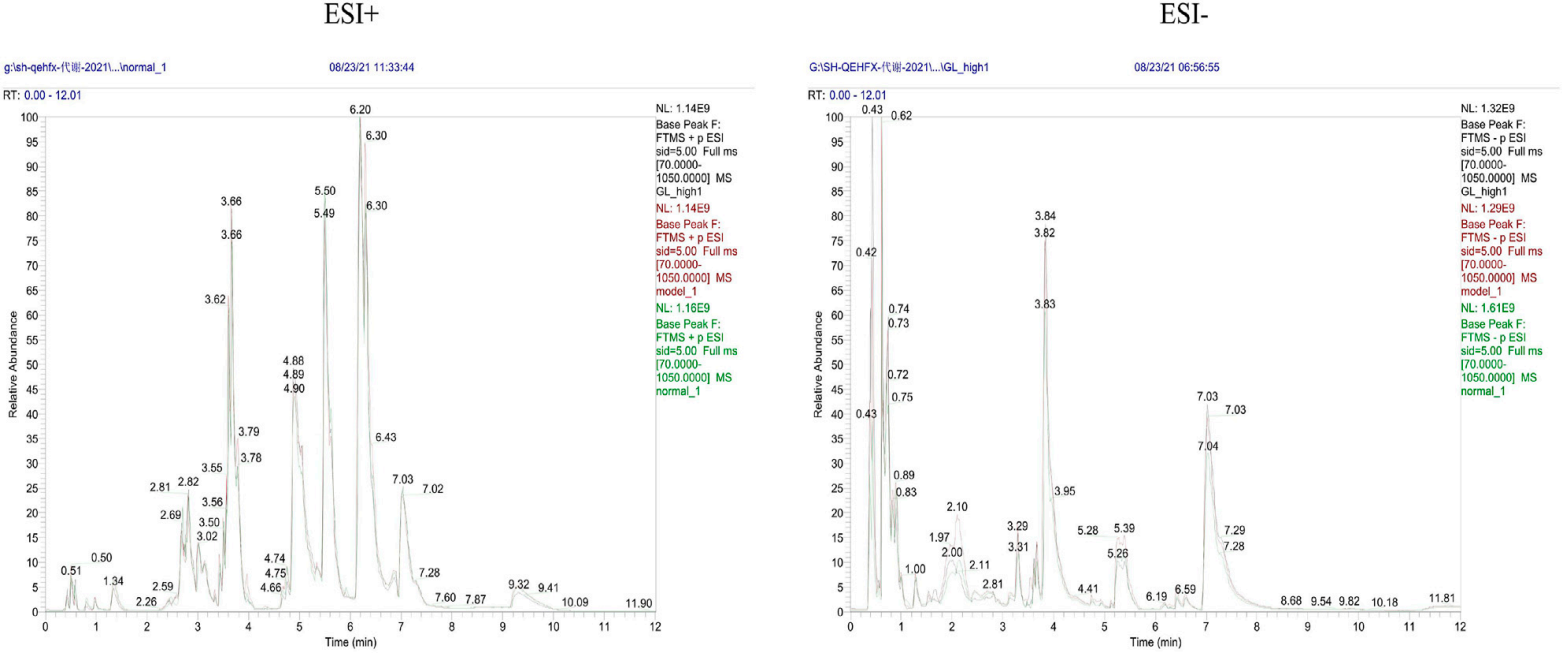
Representative BPC of rat Serum in positive and negative ion mode for each group.

**FIGURE 9:**
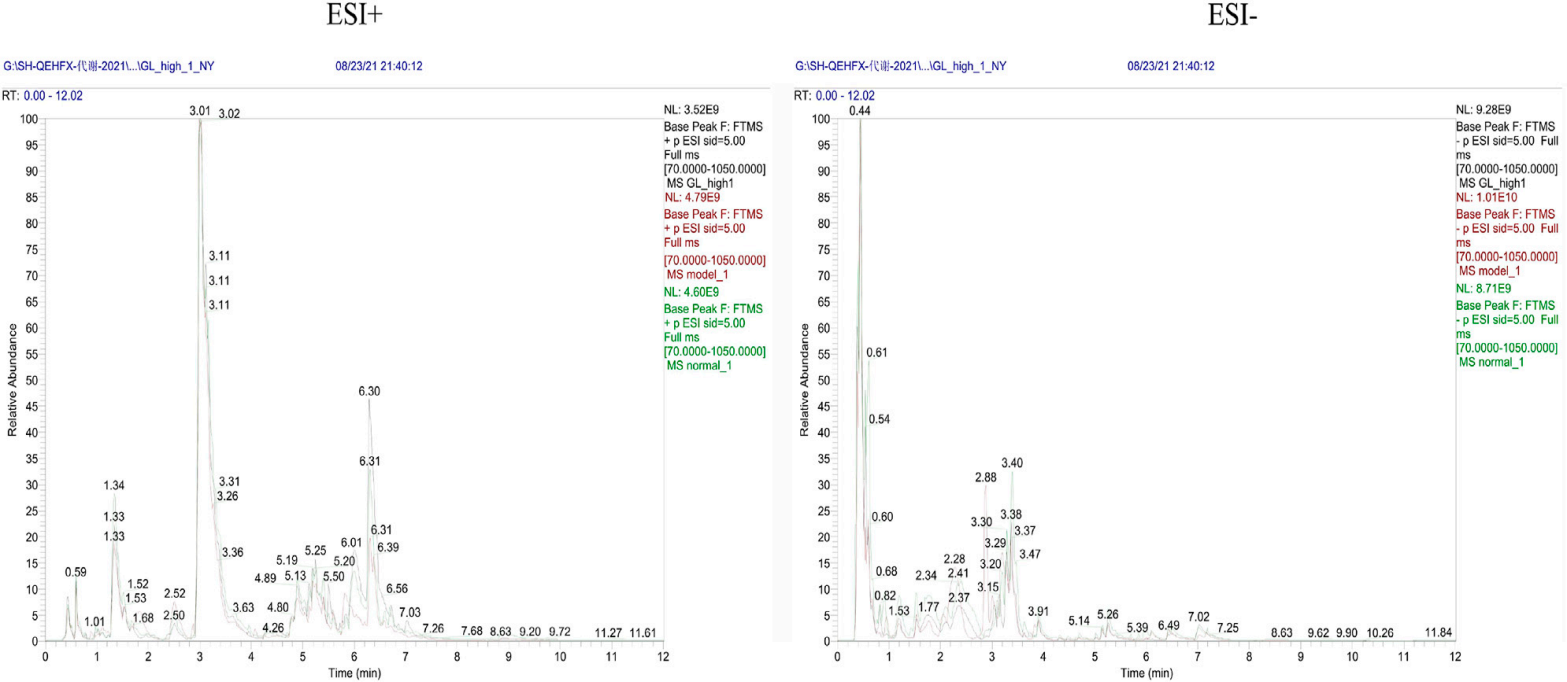
Representative BPC graph of rat urine in positive and negative ion mode for each group.

The authors apologize for this error and state that this does not change the scientific conclusions of the article in any way. The original article has been updated.

