# Supplementary figures and images for: Metabonomic analysis of the anti-hepatic fibrosis effect of Ganlong capsules
Source: Front Pharmacol. 2023 Mar 23;14:1122118. doi: 10.3389/fphar.2023.1122118 (PMC10076698; doi:10.3389/fphar.2023.1122118)

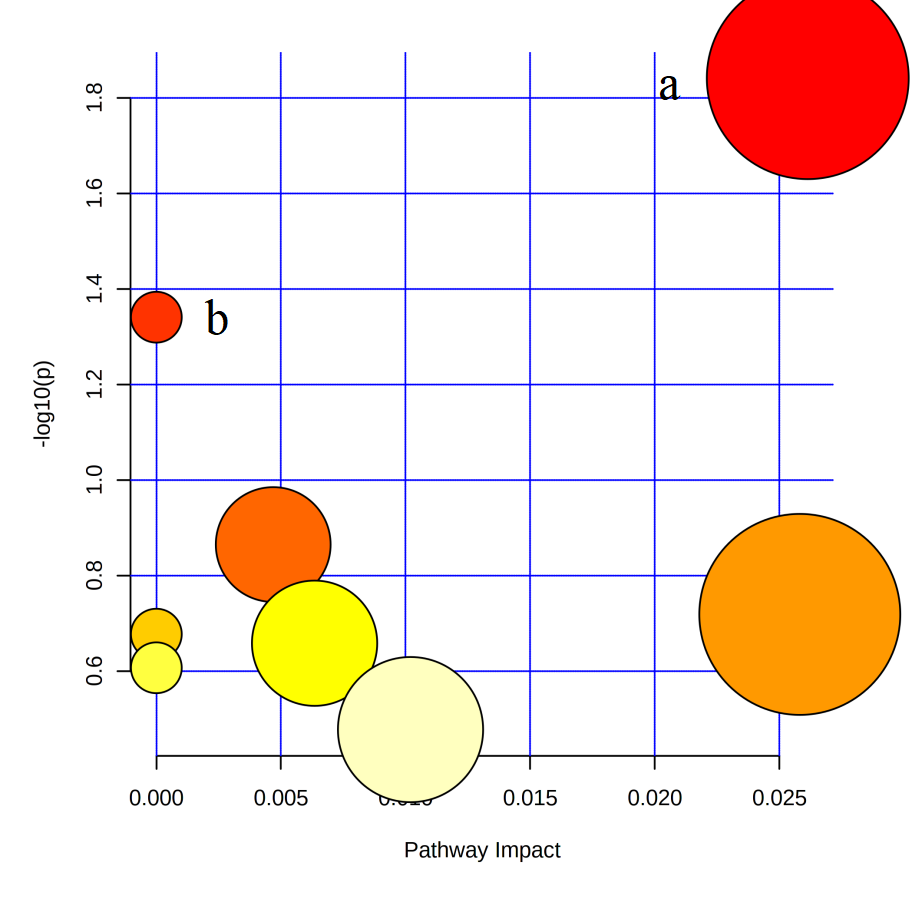

Supplement: Supplementary file 1 [file Image3.jpeg]

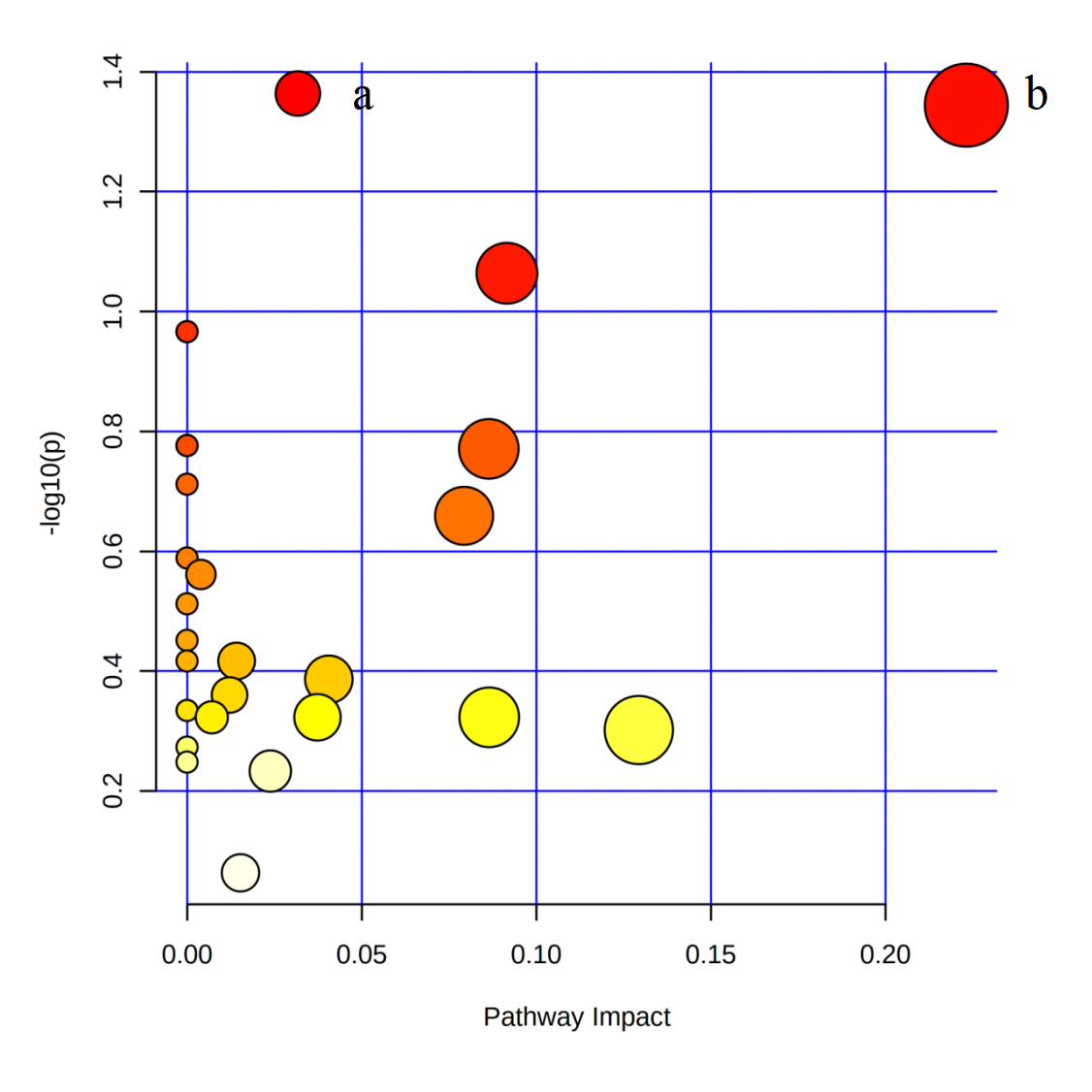

Supplement: Supplementary file 2 [file Image1.jpeg]

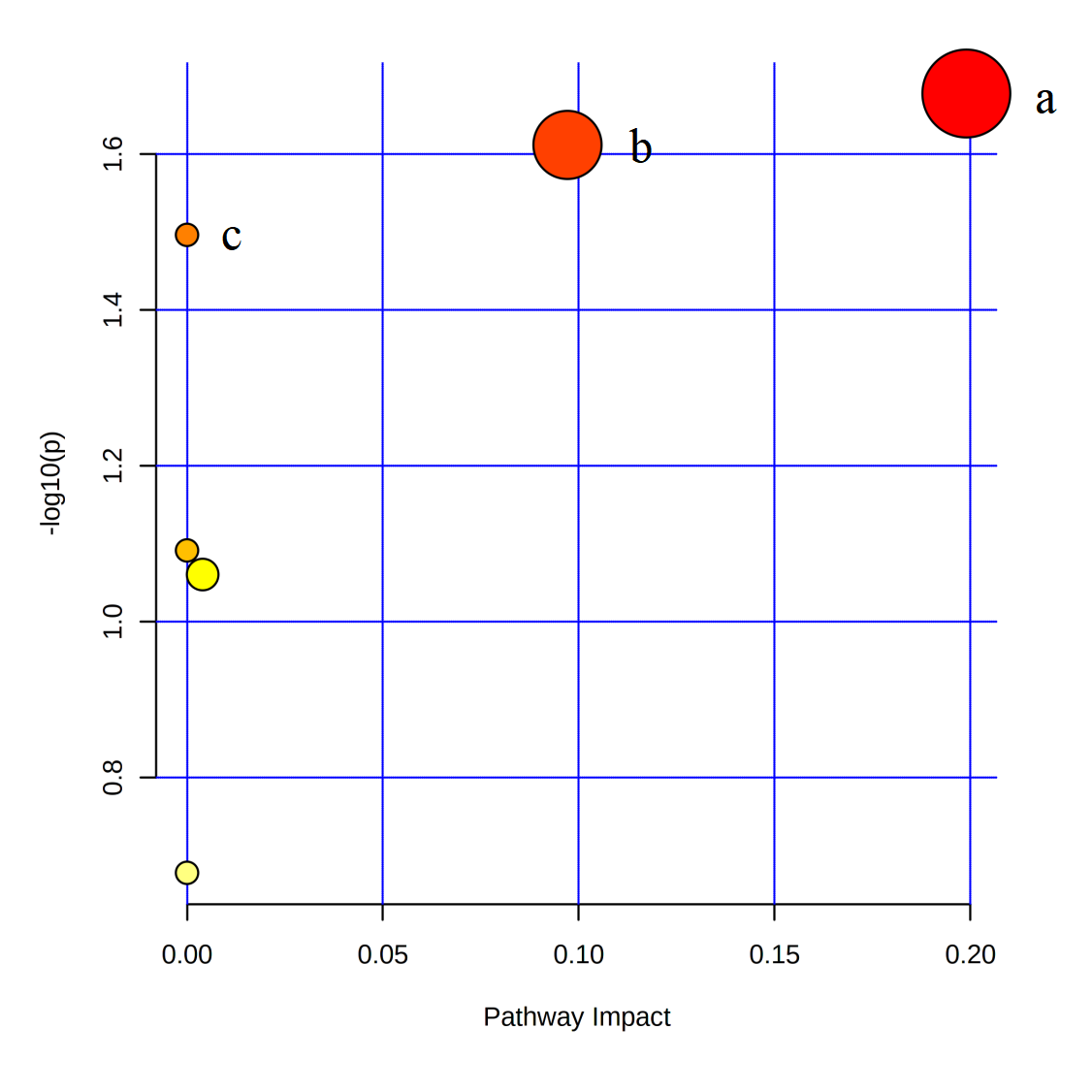

Supplement: Supplementary file 3 [file Image2.jpeg]
