# Supplementary material for: Metabonomic analysis of the anti-hepatic fibrosis effect of Ganlong capsules
Source: Front Pharmacol. 2023 Mar 23;14:1122118. doi: 10.3389/fphar.2023.1122118 (PMC10076698; doi:10.3389/fphar.2023.1122118)
Supplement: Supplementary file 4 [file DataSheet1.docx]

Table S1 Parameters of the rat liver tissue PCA model

| Model | Scan mode | A | N | R^2^X(cum) | Title |
| --- | --- | --- | --- | --- | --- |
| M1 | ESI+ | 4 | 23 | 0.559 | Total with QC |
| M2 | ESI+ | 3 | 12 | 0.599 | Model vs Control |
| M3 | ESI+ | 4 | 12 | 0.609 | Ganlong vs Model |
| M4 | ESI- | 4 | 23 | 0.588 | Total with QC |
| M5 | ESI- | 3 | 12 | 0.653 | Model vs Control |
| M6 | ESI- | 3 | 12 | 0.585 | Ganlong vs Model |

Notes: (1) Model: the model number of the multivariate statistical analysis built; (2) Scan mode: the scan mode; (3) A: the number of principal components in the modelling; (4) N: the number of samples included in the model; (5) R2X(cum): the model explanation rate; (6) Title: the data source of the model.

Table S2 Parameters of the rat liver tissue OPLS-DA model

| Model | Scan mode | A | N | R^2^X(cum) | R^2^Y(cum) | Q^2^(cum) | Title |
| --- | --- | --- | --- | --- | --- | --- | --- |
| M1 | ESI+ | 1+1+0 | 12 | 0.37 | 0.984 | 0.777 | Model vs Control |
| M2 | ESI- | 1+1+0 | 12 | 0.412 | 0.989 | 0.854 | Model vs Control |
| M3 | ESI+ | 1+1+0 | 12 | 0.313 | 0.983 | 0.578 | Ganlong vs Model |
| M4 | ESI- | 1+1+0 | 12 | 0.333 | 0.98 | 0.614 | Ganlong vs Model |

Notes: (1) Model: the model number of the multivariate statistical analysis established; (2) Scan mode: the scan mode; (3) A: the number of principal components in the modelling; (4) N: the number of samples included in the model; (5) R2X(cum): the cumulative explanatory rate in the X-axis direction of the model; (6) R2Y(cum): the cumulative explanatory rate in the Y-axis direction of the model; (7) Q2(cum): the predictive power of the model; (8) Title: the data source of the model.

Table S3 Parameters of the rat serum PCA model

| Model | Scan mode | A | N | R^2^X(cum) | Title |
| --- | --- | --- | --- | --- | --- |
| M1 | ESI+ | 4 | 23 | 0.578 | Total with QC |
| M2 | ESI+ | 3 | 12 | 0.528 | Model vs Control |
| M3 | ESI+ | 3 | 12 | 0.533 | Ganlong vs Model |
| M4 | ESI- | 3 | 23 | 0.545 | Total with QC |
| M5 | ESI- | 3 | 12 | 0.578 | Model vs Control |
| M6 | ESI- | 3 | 12 | 0.518 | Ganlong vs Model |

Table S4 Parameters of the rat serum OPLS-DA model

| Model | Scan mode | A | N | R^2^X(cum) | R^2^Y(cum) | Q^2^(cum) | Title |
| --- | --- | --- | --- | --- | --- | --- | --- |
| M1 | ESI+ | 1+1+0 | 12 | 0.304 | 0.98 | 0.496 | Model vs Control |
| M2 | ESI- | 1+1+0 | 12 | 0.339 | 0.981 | 0.577 | Model vs Control |
| M3 | ESI+ | 1+1+0 | 12 | 0.239 | 0.984 | 0.375 | Ganlong vs Model |
| M4 | ESI- | 1+1+0 | 12 | 0.243 | 0.988 | 0.404 | Ganlong vs Model |

Notes: (1) Model: the model number of the multivariate statistical analysis established; (2) Scan mode: the scan mode; (3) A: the number of principal components in the modelling; (4) N: the number of samples included in the model; (5) R2X(cum): the cumulative explanatory rate in the X-axis direction of the model; (6) R2Y(cum): the cumulative explanatory rate in the Y-axis direction of the model; (7) Q2(cum): the predictive power of the model; (8) Title: the data source of the model.

Table S5 Parameters of the rat urine PCA model

| Model | Scan mode | A | N | R^2^X(cum) | Title |
| --- | --- | --- | --- | --- | --- |
| M1 | ESI+ | 3 | 23 | 0.624 | Total with QC |
| M2 | ESI+ | 3 | 12 | 0.733 | Model vs Control |
| M3 | ESI+ | 3 | 12 | 0.695 | Ganlong vs Model |
| M4 | ESI- | 4 | 23 | 0.651 | Total with QC |
| M5 | ESI- | 2 | 12 | 0.704 | Model vs Control |
| M6 | ESI- | 3 | 12 | 0.616 | Ganlong vs Model |

Notes: (1) Model: the model number of the multivariate statistical analysis built; (2) Scan mode: the scan mode; (3) A: the number of principal components in the modelling; (4) N: the number of samples included in the model; (5) R2X(cum): the model explanation rate; (6) Title: the data source of the model.

Table S6 Parameters of the rat urine OPLS-DA model

| Model | Scan mode | A | N | R^2^X(cum) | R^2^Y(cum) | Q^2^(cum) | Title |
| --- | --- | --- | --- | --- | --- | --- | --- |
| M1 | ESI+ | 1+1+0 | 12 | 0.618 | 0.967 | 0.825 | Model vs Control |
| M2 | ESI- | 1+1+0 | 12 | 0.645 | 0.962 | 0.709 | Model vs Control |
| M3 | ESI+ | 1+1+0 | 12 | 0.566 | 0.963 | 0.734 | Ganlong vs Model |
| M4 | ESI- | 1+1+0 | 12 | 0.531 | 0.926 | 0.593 | Ganlong vs Model |

Notes: (1) Model: the model number of the multivariate statistical analysis established; (2) Scan mode: the scan mode; (3) A: the number of principal components in the modelling; (4) N: the number of samples included in the model; (5) R2X(cum): the cumulative explanatory rate in the X-axis direction of the model; (6) R2Y(cum): the cumulative explanatory rate in the Y-axis direction of the model; (7) Q2(cum): the predictive power of the model; (8) Title: the data source of the model.
